# Comparison of the gene expression profile of undifferentiated human embryonic stem cell lines and differentiating embryoid bodies

**DOI:** 10.1186/1471-213X-5-22

**Published:** 2005-10-05

**Authors:** Bhaskar Bhattacharya, Jingli Cai, Youngquan Luo, Takumi Miura, Josef Mejido, Sandii N Brimble, Xianmin Zeng, Thomas C Schulz, Mahendra S Rao, Raj K Puri

**Affiliations:** 1 Laboratory of Molecular Tumor Biology, Division of Cellular and Gene Therapies, Center for Biologics Evaluation and Research, Food and Drug Administration, Bethesda, MD 20892, USA; 2 Laboratory of Neuroscience, National Institute on Aging, Baltimore, Maryland 21224, USA; 3 Bresagen Inc., Athens, GA, USA; 4 National Institute of Drug Abuse, National Institutes of Health, Bethesda, MD 20892, USA

## Abstract

**Background:**

The identification of molecular pathways of differentiation of embryonic stem cells (hESC) is critical for the development of stem cell based medical therapies. In order to identify biomarkers and potential regulators of the process of differentiation, a high quality microarray containing 16,659 seventy base pair oligonucleotides was used to compare gene expression profiles of undifferentiated hESC lines and differentiating embryoid bodies.

**Results:**

Previously identified "stemness" genes in undifferentiated hESC lines showed down modulation in differentiated cells while expression of several genes was induced as cells differentiated. In addition, a subset of 194 genes showed overexpression of greater than ≥ 3 folds in human embryoid bodies (hEB). These included 37 novel and 157 known genes. Gene expression was validated by a variety of techniques including another large scale array, reverse transcription polymerase chain reaction, focused cDNA microarrays, massively parallel signature sequencing (MPSS) analysis and immunocytochemisty. Several novel hEB specific expressed sequence tags (ESTs) were mapped to the human genome database and their expression profile characterized. A hierarchical clustering analysis clearly depicted a distinct difference in gene expression profile among undifferentiated and differentiated hESC and confirmed that microarray analysis could readily distinguish them.

**Conclusion:**

These results present a detailed characterization of a unique set of genes, which can be used to assess the hESC differentiation.

## Background

Embryonic stem cells (hESC) have been isolated from multiple species [1-4] including non-human primates [2] and humans [3,4]. Currently, over hundred different Human embryonic stem cell (hESC) lines have been established [3-7]. hESC populations grow as tightly compacted colonies of undifferentiated cells on mouse [3,4] or human [6] feeders or as colonies in feeder-free conditions using matrix and conditioned medium [8]. hESC has been shown to differentiate in vitro and in vivo to form derivates of all three germ layers. In vitro differentiation can be induced by the process of embryoid body (hEB) formation, which involves aggregating the cells and preventing separation by plating on a non-permissive substrate. Cell to cell interaction and addition of differentiation agents such as retinoic acid (RA) induces differentiation into derivatives of all three germ layers (mesoderm, ectoderm and endoderm) [7,9-12].

hEB can subsequently be induced to undergo further differentiation to generate a variety of cell types, including hematopoietic [13], neuronal [14,15], myogenic [16] and cardiac muscle cells [17,18]. Thus, hEB represent an early stage in the process of lineage specification and should differ from hESC or their more differentiated progeny in their profile of gene expression.

Several different methods have been developed that can be used to assess the process of differentiation. Subtractive hybridization [19,20], differential display polymerase chain reaction (DD-PCR) [21], representational difference analysis (RDA) [22], analysis of expressed sequence tag (EST) [23] and serial analyses of gene expression (SAGE) [24] are but a few commonly used techniques. Perhaps the most commonly used however, is gene array (microarray) [25-27]. Microarrays have been used by several investigators to assess the undifferentiated hESC state [28,29] and provide a data set of useful information. We for example utilized a large-scale oligonucleotide based array to identify a set of 92 genes that are highly upregulated in six hESC lines when compared against human universal reference RNA derived from mature tissues [28]. This set of "stemness genes" along with additional novel genes identified has served to assess the state of undifferentiated cells. However, currently no similar data set is available for genes that may be used to define the embryoid body stage of hESC differentiation and no comparisons between the undifferentiated and differentiated populations have been performed utilizing microarray technology. A recent study has characterized gene expression in embryoid bodies by massively parallel signature sequencing (MPSS) and suggested that several candidate genes specific to hEB may exist [30]. MPSS analysis however, is expensive and unavailable for most laboratories. Therefore, an alternate readily available and economical assay is needed to characterize embryoid bodies and compare them with available datasets on hESC. Microarray studies of hEB's offer the possibility of such an assay that can be used for routine assessment of the state of ES cell differentiation.

To determine if microarrays (or gene arrays) can be used to distinguish between hESC and hEB and to identify candidate markers of the process of differentiation we have compared the gene expression pattern of undifferentiated hESC and differentiated hEB derived from them using a large-scale oligonucleotide based arrays. The expression of selected genes was confirmed by another large-scale array, reverse transcriptase-polymerase chain reaction (RT-PCR), comparison with an expressed sequence tag (EST) enumeration database of ESC [23], MPSS data from embryoid bodies [30] and immunocytochemistry. Our results show that microarray studies can readily distinguish between hESC and hEB and can be used to identify markers of the embryoid body stage.

## Results

### Microarray detects differences between hESC and hEB derived from them

**Figure 1:**
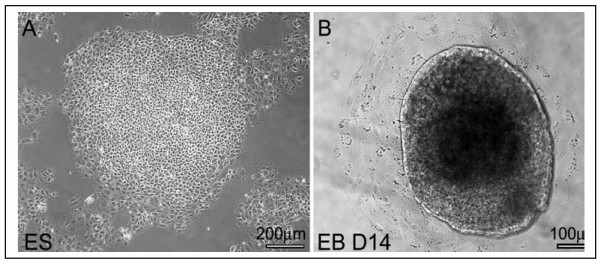
Phase differentiation of ES and EB. Phase pictures of a human ES cell clone grown under feeder-free conditions (A) and a human EB of day 14 (B).

**Figure 2:**
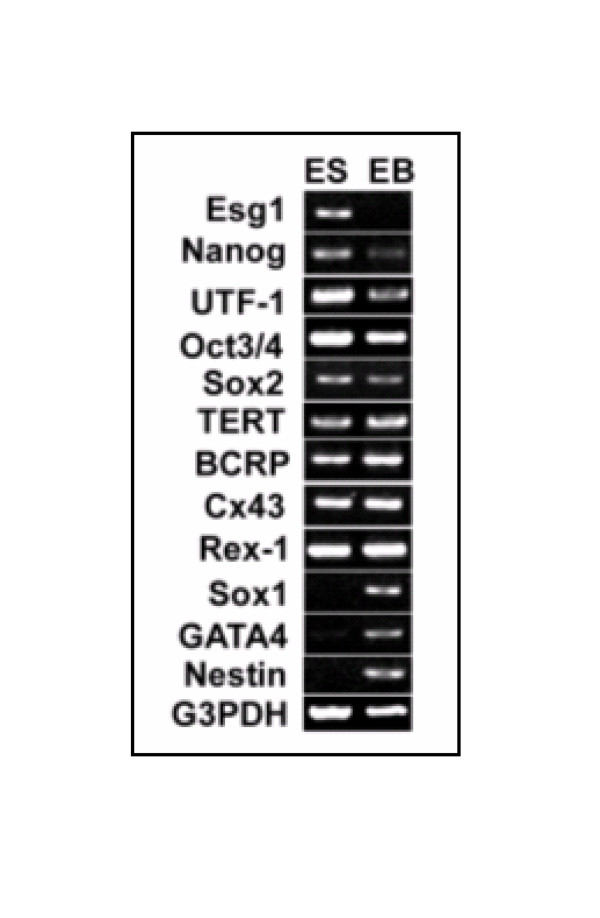
RT-PCR of some marker genes in pooled samples of ES and EB RNA. RT-PCR analysis of marker genes in Pooled ES and pooled EB cell lines. Total RNA derived from both the ES and EB cell lines (WiCell lines) were subjected to RT-PCR analysis as previously described (34). G3PDH mRNA amplified from these samples served as an internal control. The primers used are listed in supplementary table (Table 7S). The thermocycler conditions used for amplification were 94°C, 4 minutes hot start,94°C, 30 sec; 60°C, 30 sec and 72°C, 1 minute. Ten microliter amplification products were resolved in 2% agarose gel, stained with ethidium bromide (EtBr), and visualized in a transilluminator and photographed.

To assess alteration in gene expression in hESC and hEBs, cells were cultured and induced to form EBs (Fig. 1). Total RNA was harvested from undifferentiated hESCs and hEBs derived from them and a time course of gene expression was performed to assess downregulation of known ES cell specific genes. Day 13 of differentiation was chosen as a time point for subsequent analysis where there is a clear downregulation of known hESC markers and an upregulation of some early markers of differentiation (Fig. 2). The expression of known undifferentiated hESC markers including Oct4, Nanog and Esg1 showed reduction in expression in hEB at day 13 while known markers of differentiation (SOX1, Nestin, and GATA4) showed a marked increase in expression. Several genes (Sox2, TERT, BCRP, Cx43, and Rex-1) did not show detectable change between hESC and hEB cells. For microarray studies each sample was compared to human universal reference RNA (mixture of total RNAs from a collection of adult human cell lines, chosen to represent a broad range of expressed genes and both male and female donors are represented) to maintain uniformity and allow comparisons across samples. cDNA from BG02 and pooled samples of hESC were labeled with Cy5 and huURNA with Cy3, and ~17,000 oligonucleotide arrays were hybridized. Similarly, cDNA from hEB derived from differentiated BG02-ESC (day 13 BG02 EB, day 21 BG02 EB) and pooled WiCell EB (day 13) was labeled with Cy5 and huURNA with Cy3 and arrays were hybridized and data analyzed. Since the process of differentiation is relatively stochastic and cell lines may behave differently as they differentiate, data from different experiments (with different samples) was not pooled and reported as the expression from single hybridization. Each sample was analyzed in duplicate obtained from two different independent cultures.

Overall microarray results between technical replicates were similar and representative images from each experiment are available (see Additional file-1, 2, 3, 4, 5). Microarray analysis showed that 11,000 of the ~17,000 features present on the array were detectable above background at a ≥ 150 minimum intensity and target pixels of one standard deviation (SD) above background (≥30) cutoffs. hEB and hESC expressed approximately 2400 to 3000 genes. Pooled WiCell ESC and BG02 ESC showed over expression of 2471 and 2843 genes at ≥ 2 fold respectively, compared to huURNA (Supplementary Table-1Sa and 1Sb, see additional file-6 and 7). As these cells differentiated to hEB, the number of total genes that were detected remained similar to hESC (supplementary Table 2S, see additional file-8).

For some experiments ESC samples were pooled as we were interested in identifying differences between ESC and EBs that would be common to multiple lines. Further, as EB formation is variable we felt pooling may allow us to focus on large differences which would not be lost in the averaging process. Once we obtained results we then tested if this was true by using cell line provided by a different provider and propagated under different culture condition. In addition, since the purpose of day 21 differentiation was to study which genes persisted and which were modulated as a result of further differentiation we did not use day 21 pooled EBs but focused on results from a single line

We have previously shown that six hESC lines (BG02, BG01, GE01, GE09, TE06 and PES cell lines from GE01, GE07 and GE09) express 92 genes in common at ≥ 3 fold levels when compared with huURNA (28). We therefore examined the expression of these genes and in addition all genes overexpressed in BG02 and pooled hESCs (Table 1/see additional file-9) (supplementary Table 1Sa and 1Sb, see additional file 6 and 7). Eighty-seven of 92 genes were also detected in the present sample confirming the quality of the array hybridization and the fidelity of the samples used. In addition, previous study identified several early markers of differentiation, that were present at low levels in hESC and when hESC cells were differentiated, these genes were upregulated and up regulation was confirmed by EST enumeration technique [28]. The present study confirmed these earlier observations by microarray experiments (Table2/see additional file 9). These differentiation genes included Keratin 8, Keratin 18, ACTC, and TUBB5. When we examined our current results, we found that these genes were also up regulated in hEB cells derived from BG02 and WiCell lines by microarray studies (Table 2/ see additional file 9). These results confirm the EST enumeration data and further provide support for the quality of hEBs used.

Overall the technical replicates, the testing of the expression of known genes and the ability to detect expected changes in the current samples confirmed the suitability of the current data set for additional analysis.

### Modulation of "stemness genes by ES cell differentiation

Previously, we identified a set of 92 genes are expressed at high levels in most hESC and can be detected by microarray and EST enumeration [28]. To test if changes in their expression could be used to monitor differentiation, we examined the relative levels of these genes as ES cell differentiated. Eighty-seven of the 92 genes expressed in all six hESC lines were also over expressed in day 13 BGO2-EB and day 13 pooled WiCell EB compared to HuURNA (data not shown). However, as a result of differentiation of the BG02-ESC line 77 out of 92 genes were down modulated in BG02 EB cells (Table 1/ see additional file 9). Among these, known ES cell markers e.g., POU5F1, GTCM-1, LEFTB, Galanin, GJA1, TDGF1, SFRP2, FABP5 and Lin-28 showed a marked decline in their expression compared to undifferentiated ES cells. Similarly, other ES cell specific markers such as CER1 and DNMT3B also showed marked decline in the expression in day 13 BG02 EB compared to ESC (Table1/ see additional file 9). However, three zinc finger proteins did not show any significant change and down modulation of Numatrin, C20orf129 and Laminin receptor was modest (Table1/ see additional file 9).

Twelve novel genes that were overexpressed in all 6 hESC lines tested [28], were all down modulated by differentiation. Among them MGC27165 (IFITM1), GSH1, PPAT, KIAA1573, TD-60, C20orf168, ARL8 showed a marked decline in fold expression in BG02 EB compared to undifferentiated BG02 (Table 1/ see additional file 9) while changes in other genes was modest.

Pooled EB samples of ES cells (WA01, WA07 and WA09) showed a similar gene expression profile as observed in day 13 BG02-EB. 53 out of 92 "stemness" genes were down modulated compared to undifferentiated WiCell ESC (Table 1/ see additional file 9). These 53 genes were a subset of the 77 that were markedly downmodulated in BG02 derived EB compared to undifferentiated BG02-ESC (see above and Table 1/ see additional file 9). POU5F1, LEFTB, FABP5, Galanin, GJA1, SFRP2, Nanog and TDGF1 ES specific genes showed ≥ 3 times lower expression in pooled WiCell EB compared to pooled WiCell ESC (Table 1/ see additional file 9). In addition, other ES cell specific markers like DNMT3B, CER1, and SOX2 also showed down modulation in pooled WiCell EB. Novel genes such as PPAT, MGC27165, GSH1, ARL8, and KIAA1573 showed marked down modulation in pooled EB compared to pooled ES cells. However, zinc finger protein Znf257, laminin receptor, C20orf1 and C20orf129 showed modest decline (Table 1/ see additional file 9).

Both pooled WiCell EB and day 13 BG02-EB showed an upregulation of genes that we had previously identified as being markers of differentiation that were present in detectable levels in undifferentiated hESC lines (Table 2/ see additional file 9). These included early differentiation markers e.g., KRT8, KRT18, TUBB5 and ACTC. Overall, the pattern of differentiation appeared similar with BG02 when compared to embryoid bodies derived from WiCell ESC.

Among 92 genes, thirteen additional novel genes have been identified previously in hESC [28]. However, only 3 of the thirteen genes were present on this array and their expression was analyzed. Our results show that Dppa4 was expressed in BG02 ES and pooled WiCell ES, however, its expression level was eight fold higher in day 13 BG02 EB compared to huURNA and down modulated to 2 fold at day 21 BG02-EB. Similarly, pooled WiCell hESC cells showed 10 fold over expression of Dppa4 compared to HuURNA, however upon differentiation to EB the expression was decreased. In sharp contrast, claudin-6 showed down modulation of expression from pooled WiCell ESC to EB by about 10 fold but was not present in detectable levels in either BG02 ES or BG02 EBs.

In PEB some genes that showed down modulation in BG02 EBs were in fact up regulated compared to ESC. The reason for this reverse pattern is not known. Although we did not check whether these were also up regulated in day 21 pooled EBs, our future studies are focused on addressing these issues. It is possible that a mixture of three hESC lines behaved differently when they were mixed and differentiated.

Overall, these data confirm that seventy-seven of the ninety-two genes we had identified as hESC specific could be used to assess the differentiation state of ES cells as they differentiate. A large number of these genes show a dramatic down modulation as early as Day 13 and this is seen in both WiCell and Bresagen (BG02) cell lines and can be readily assessed by microarray analysis.

### Genes that are upregulated as ES cells differentiated to form embryoid bodies

To analyze genes that were upregulated as hESC differentiated, embryoid bodies were prepared as previously described and their quality analyzed as described above. Samples, which showed a clear downmodulation of ES cell specific genes, were subjected to microarray studies and their overall gene expression pattern compared with that of the undifferentiated population. Each individual experiment was analyzed separately and differences in gene expression observed were then compared. When BG02 hESC were compared with embryoid bodies derived from them a total of 333 new genes were expressed at ≥ 3 fold higher levels in day 13 BG02-EB compared to undifferentiated BG02 ESC (Supplementary Table 3S, see additional file-8). These genes included many genes known to be upregulated in EB cells and confirmed the quality of the array and the hybridization (see supplementary data). In addition, we identified numerous additional genes whose expression in hEBs had not been documented previously.

Since we were interested in identifying a core set of genes that are upregulated as ES cells differentiated we compared the pattern of gene upregulation in a second distinct population of ES cells (WiCell lines) grown under different conditions using protocols described previously [7,31,32]. Out of 333 genes that were upregulated in day 13 BG02 EB 194 genes were also upregulated at ≥ 3 fold in WiCell derived EB (pooled EB) (Table 3/ see additional file 9). The remaining 139 genes were also upregulated but they did not meet the rigorous three fold cutoff criteria. The expression of these 139 genes by other techniques will be subject of confirmation in future studies.

**Figure 3:**
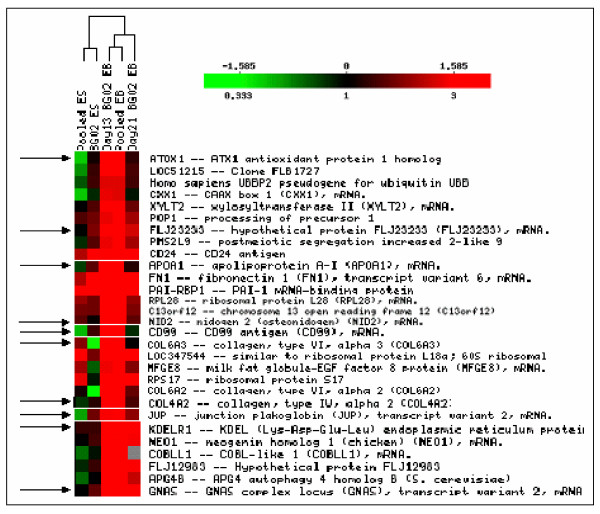
Hierarchical clustering of a set of genes over expressed in EB but not in ES. Hierarchical clustering of genes overexpressed in EB but not in ESC. All 194 genes were clustered; however, a snap shot of some genes is shown due to space limitation. A set of genes showing overexpression at ≥ 3 fold cluster together in BG02-EB and pooled EB but differ from BG02-ESC and pooled ESC. Color indicates the relative expression levels of each gene, with red indicating higher expression, green indicating negative expression and black representing absent expression. The 10 genes as indicated by the arrows can be considered as marker for EB as they are either negatively expressed or absent in ESC in most cases but overexpressed in both the EBs.

The common subset of genes, which showed upregulation at ≥ 3 fold in both BG02 EB and pooled WiCell EBs were classified by hierarchical clustering. As shown in Fig 3, all listed genes (a snap shot of 194 genes) were upregulated in EBs derived either from BG02 or WiCell lines and the expression levels can be readily distinguished from those in undifferentiated ESC. These results clearly show a marked difference in gene expression profile in differentiated compared to undifferentiated ES.

More detailed examination of the genes that were differentially expressed in ESC and EBs indicated multiple signaling pathways are altered. BG02 EB showed over expression of Keratin 19, Profilin-1, Fibronectin 1, HAND1, COL1A2, ZAK, COL4A2, BIRC7, NID2, TUBB5, TMSB4X, PLP2, ENO1 and COL5A2 (Supplementary table 4S, see additional file-8). Pooled WiCell EB also showed upregulation of similar genes including HAND1, KRT19, Fibronectin 1, Profilin1, TMSB4X, Vimentin, Enolase, PLP2, COL4A2, CAPN1, NID2, IVL, ZAK, SPTA1 and COL1A2, which are related to cell differentiation or cytoskeleton (Supplementary Table 5S/see additional file 8). Several genes related to cell signaling, cell growth, cell cycle and metabolic activities were uniquely identified (see supplementary table 3S or see additional file-8) and (Table 3/ see additional file 9). For example, Glypican-3, member of the glypican-related integral membrane proteoglycan family (GRIPS) contains a core protein anchored to the cytoplasmic membrane via a glycosyl phosphatidylinositol linkage and plays a role in the control of cell division and growth regulation was over expressed. Calreticulin (CALR), which can inhibit androgen receptor and retinoic acid receptor transcriptional activities in vivo, as well as retinoic acid-induced neuronal differentiation was over expressed by 5 fold. Cyclin-dependent kinase inhibitor 1C (CDKN1C), an inhibitor of several G1 cyclin/CDK complexes (Cyclin-E-CDK2, Cyclin-D2-CDK4 and Cyclin-A-CDK2) and to lesser extent of the mitotic cyclin-B-CDC2, was over expressed in EBs. Finally, 26 hypothetical, 2 zinc finger proteins and 9 unknown genes whose functions are still to be determined were also over expressed in EBs (Supplementary Table 3S and 6S or see additional file-8) and (Table 4/ see additional file 9).

Overall, these results indicate that 194 genes identified as upregulated in two different EB cell lines may serve as early and sensitive markers to monitor ES cell differentiation.

### Confirmation of gene expression profile by microarray, MPSS, EST-enumeration, RT-PCR and immunohistochemical analyses

To provide independent verification of the results we utilized three different strategies. We compared gene expression patterns obtained using microarray studies with the MPSS data set generated by our laboratory using the pooled ES and EBs derived from them [30]. In addition, we prepared duplicate samples of BG02 ES and BG02 derived embryoid bodies and subjected them to a microarray analysis using a large scale oligonucleotide array based on a different set of oligonucleotides that were commercially available (Agilent, Foster city, CA). Finally we examined a subset of genes that were not validated by either of these methods by RT-PCR. Of the 194 genes, which were uniquely over expressed at ≥ 3 fold in both BG02 and WiCell cell derived EBs 148 genes showed higher expression in an MPSS analysis of WiCell samples (data now shown and reference [33]. Overall, comparison of microarray results with MPSS showed a high concordance in gene expression profile. For example, known ES cell specific markers including such as POU5F1, Galanin, DNMT3B, GJA1, LEFTB and TDGF1 showed higher expression in undifferentiated BG02 and PES cells compared to differentiated BG02 or PEB by both microarray and MPSS analyses (Table 5/ see additional file 9). Similarly, early ES differentiation markers e.g., KRT8, KRT18, KRT19, ACTC and EB specific genes such as Vimentin, AFP, HAND1, and COL1A2 showed higher expression in EBs compared to ESC by both microarray and EST enumeration (Table 6 and Table 7/ see additional file 9).

**Figure 4:**
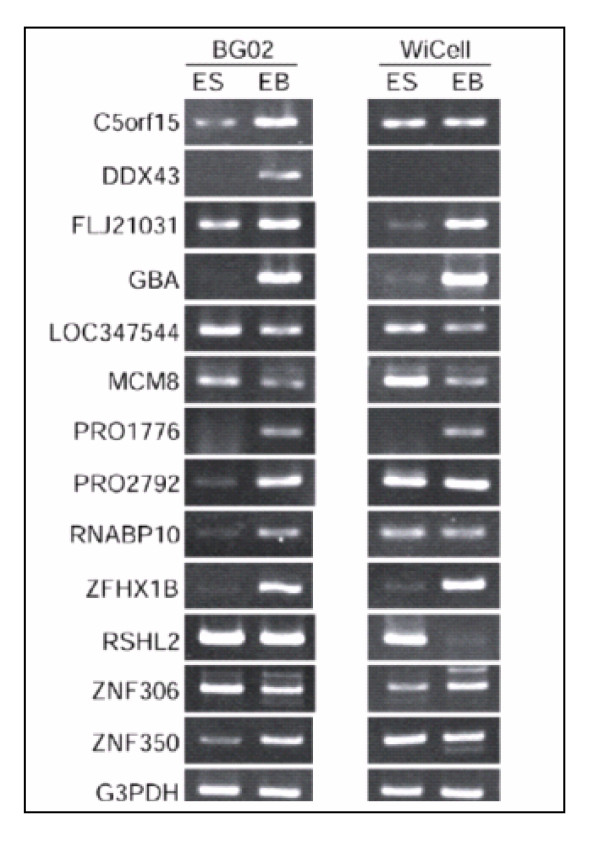
RT-PCR analysis of some overexpressed genes and novel genes in PEB and EB derived from BG02 compared to ES. RT-PCR analysis of novel genes in PEB and BG02 EB compared to respective ESC. Total RNA derived from both the ES and EB cell lines (BG02 and WiCell lines) were used and RT-PCR performed as described in Fig. 2 legend. G3PDH mRNA served as an internal control.

**Figure 5:**
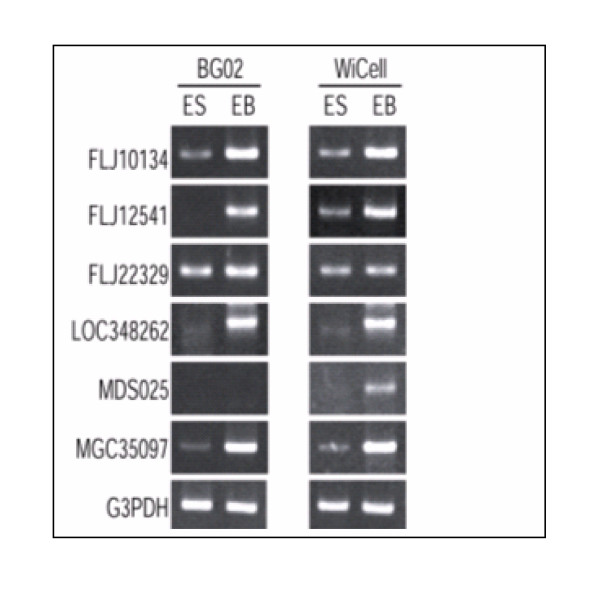
RT-PCR analysis of some novel genes over expressed in PEB and EB derived from BG02 compared to ES. RT-PCR analysis of six novel genes in EB confirmed by MPSS. Total RNA derived from both the ES and EB cell lines (BG02 and WiCell lines) were subjected to RT-PCR analysis as described in Fig. 2 legend. G3PDH mRNA amplified from these samples served as an internal control.

**Figure 6:**
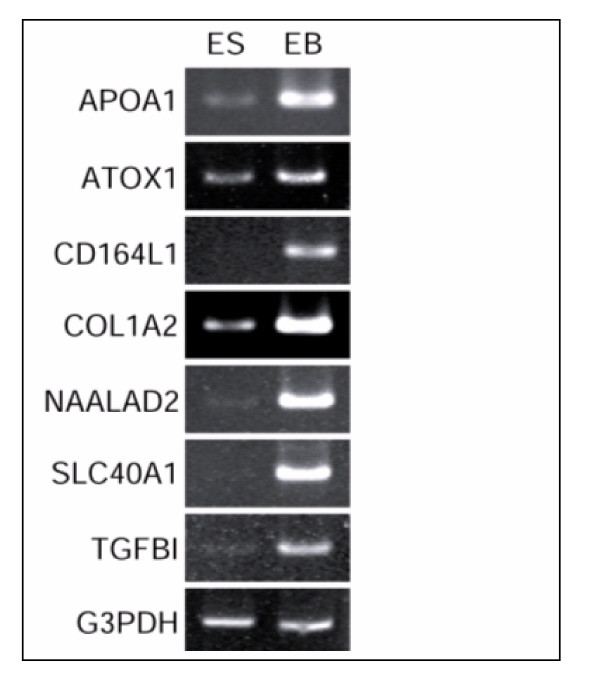
RT-PCR analysis of some distinctly over expressed genes in EB. RT-PCR analysis of some distinctly overexpressed genes in EB as identified by microarray analysis. Marked overexpression in EB is clearly documented in the figure. Total RNA derived from both the ES and EB cell lines (BG02 and WiCell lines) were subjected to RT-PCR analysis as described in Fig. 2 legend. G3PDH mRNA amplified from these samples served as an internal control.

When fold expression of 8 out of 9 unknown genes identified by microarray was compared with tpm level detected by MPSS, a similar pattern of gene expression was observed (Table 8/ see additional file 9). All of these genes showed higher expression in BG02-EB and WiCell derived EB compared to BG02-ES and WiCell ESC samples. Six of these genes were also confirmed by RT-PCR analysis (Fig. 5). The reliability of microarray results was further confirmed by RT-PCR analysis of 10 genes for hypothetical proteins, 2 for zinc finger proteins, 3 for unknown proteins and 7 genes that were highly expressed in embryoid bodies (Fig. 4, 5, 6). This similarity in the results confirmed the reliability of the microarray studies.

As forty-six of the 194 genes (Table 9/ see additional file 9) identified by microarray showed no significant difference by MPSS analysis, it was concluded that microarray and MPSS assays may be different in sensitivity or there was variability in the production of embryoid bodies. To address this issue and to determine if the differences observed by microarray were reliable, we used a second microarray platform to examine gene expression in the same samples. Interestingly 33 of the 46 genes showed overexpression in WiCell derived EBs using Agilent human 22 k oligo-arrays (Table 9/ see additional file 9). This suggested that different methods have different sensitivities and it is important to use multiple methods to confirm expression.

The expression of the remaining 13 genes detected as overexpressed by microarray studies using a custom built microarray but not by MPSS or by Agilent microarrays was tested by RT-PCR analysis (Fig 4). Of the thirteen genes, 9 were confirmed by RT-PCR analysis further confirming the differential sensitivity of various arrays and other large-scale analytical methods.

### Gene expression profile of Day 21 BG02-EB

Our results identified a large set of genes that are differentially modulated as cells differentiate to form embryoid bodies over a period of two weeks. To examine whether the same set of genes could be used to assess differentiation of hESC at twenty-one day of differentiation, we examined gene expression using RNA prepared from Day 21 embryoid bodies. For these studies, undifferentiated hESC (BG02-ESC) cells were used to generate EBs by a brief exposure to collagenase IV and small clusters of cells were obtained by scraping with a pipette. ES cells were differentiated for 13 and 21 days, harvested to prepare total RNA and analyzed by hybridization to microarrays. We found that a majority of ESC specific genes that were down modulated as cells differentiated for thirteen days were further down modulated as a result of differentiation to day 21 BG02-EB (data not shown). Among 194 genes overexpressed in day 13 BG02-EB and PEB, thirty-three genes showed a decrease in fold expression at day 21 BG02-EB. However, other than COL1A2, which was down modulated from 27 fold to 3 fold, none showed any marked decrease. The remaining genes were either not expressed or did not show any change in the expression (data not shown).

We previously reported that eight early differentiation marker genes were expressed in hESC, which were further upregulated in hEBs as determined by EST enumeration [28]. Therefore, in this study we examined their expression at day 21 of differentiation. For this experiment gene expression between BG02 ES was compared with day 13 BG02-EB and day 21 BG02-EB. We found that six of these genes were markedly down modulated in day 21 BG02-EB (Table 10/ see additional file 9) compared to day 13EB and PEB. The other two genes showed minor or no significant change.

In addition, expression of 11 known EB specific genes that were shown to be overexpressed in both day 13 BG02-EB and pooled EB (supplementary table 4S and 5S or see additional file-8) also examined in day 21 BG02-EB. Interestingly, all of them were down modulated in day 21 BG02-EB (Supplementary table 4S or see additional file-8)

Additionally, we examined the status of genes that were upregulated in day 21EB but not in ES or day 13 BG02-EB. Genes related to cytokeratin and hair keratin (e.g., KRT17, KRT20, and IVL), which are responsible for structural integrity showed higher expression in day 21EB compared to BG02ES or day 13EB. In addition, genes related to mature tissues were exclusively upregulated in day 21EB but not in ES and day 13 BG02-EB (Supplementary Table 4S or see additional file-8) indicating that additional differentiation markers can be used to distinguish early vs. late EBs.

**Figure 7:**
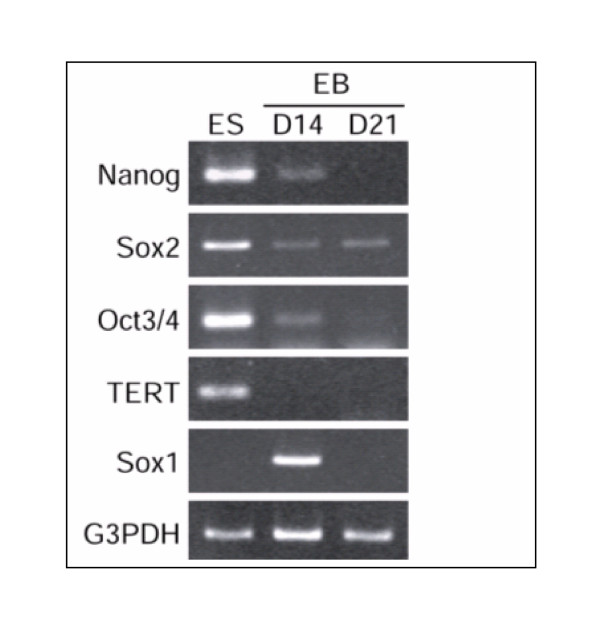
RT-PCR analysis of some ES and EB specific genes. RT-PCR analysis of ES and EB specific genes in day 14 and day 21 BG02-EB and BG02-ES samples. Total RNA derived from both the ES and EB cell lines (BG02-ES and EB) were subjected to RT-PCR analysis as described in Fig. 2 legend. G3PDH mRNA amplified from these samples served as an internal control.

**Figure 8:**
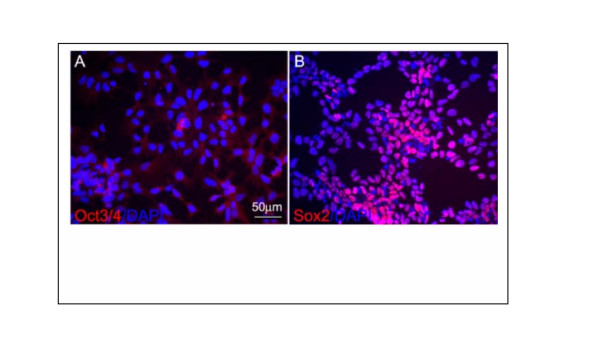
Immunocytochemistry of Oct-3/4 and Sox-2 in ES and EB. Immunocytochemistry of Oct3/4 and Sox-2 in ES and EB. Human neural progenitor cells derived from ES cells were stained with various antibodies. (A) All neural progenitor cells are Oct3/4 negative and (B) most of these cells are positive for Sox2. DAPI staining shows every cell in (A) and (B).

While most genes followed an expected pattern of change in expression in day21 EB, Nanog showed a reverse pattern of expression. On day 13 its expression level was significantly reduced compared to BG02-ES, however, on day 21EB an increased expression was observed compared to day 13 BG02-EB. Levels were almost similar to those in BG02-ESC samples (data not shown). Downmodulation of Nanog in day 13-EB was confirmed by RT-PCR analysis (Fig 7) though its upregulation in Day 21 samples did not match the RT-PCR results (see discussion). The downregulation in nanog followed the downregulation of Oct 3/4 and TERT and other ES specific genes (Figure 7 and data not shown). In addition, although Sox 2 gene showed a decrease in expression in pooled EB compared to pooled ESC but it didn't show any change in expression pattern from BG02ES to day 13 BG02-EB and day 21 BG02-EB (data not shown). Expression of Sox-2 gene was also confirmed by RT-PCR and immunocytochemistry analyses (Fig.7 and Fig. 8). Both analyses demonstrated that Sox2 is expressed in day13 and day 21EBs. Oct3/4 protein expression was used as a control that did not show expression in Sox-2 expressing cells confirming that the Sox-2 expression represented induction in a newly differentiated population rather than in persistent ESC.

These results indicate that KRT8, KRT18, TUBB5, ACTC, SERPINH1, TUBB4, KRT19, HAND1, FN1, ENO1, COL1A2, COL5A2, COL4A2 and other identified markers may serve as a indicators of early and late stages of differentiation as they were further markedly down regulated on day 21 compared to day 13 of differentiation. In contrast, genes such as KRT17, KRT20, IVL, NPHP3, CAPN1, and CNTN6 are markers of later stage of differentiation as they are overexpressed in day 21 compared to day 13 of differentiation. Thus microarray studies can distinguish between ES cells and embryoid bodies as well as between early and late stage embryoid bodies.

## Discussion

Our results show that large-scale oligonucleotide based microarrays can be used to distinguish between undifferentiated hESC and differentiating hEBs. Two sets of markers can be distinguished in a set of 'stemness" genes that are present at higher levels in undifferentiated cells whose levels are reduced as cells differentiate and a complementary set of genes that are absent or present at low levels in hESC and are upregulated as cells differentiate. The set of genes that are upregulated as cells differentiate include known as well as unknown genes. This pattern of gene expression was confirmed by a variety of independent means including microarray using a second independent array set, comparison with MPSS using similar samples and by RT-PCR. The combined set of upregulated and down regulated genes will serve as a sensitive indicator of the state of hESC and the novel sets of genes identified are likely candidates that may participate in regulating the process of differentiation.

In our previous study we demonstrated over expression of a set of 92 genes in six undifferentiated ESC cell [28]. Expression of almost all of the genes was confirmed in the BG02-ES and pooled hESC used in the present study. The present study further refines the set of stemness genes to identify those that are rapidly down-regulated as ES cells differentiate. We show that 77 of these are down modulated when differentiated to embryoid bodies using undifferentiated BG02-ES. Further differentiation of BG02-ES to day 21 BG02-EB showed consistency in down modulation of many of these. This refined subset of previously identified stemness genes can be used to monitor the transition of undifferentiated pluripotent human embryonic stem cells to differentiated embryoid bodies. Analyzing the pattern of gene expression, we would suggest that ES specific genes such as Lin-28, PSIP2, PITX2, DNMT3B, and Galanin whose downregulation is seen in both lines and confirmed by EST scan and microarray represent good initial candidates to assess the ES cell state. Furthermore, LEFTB and CER1, inhibitors of nodal signaling, which were downmodulated as ES cells differentiated (data not shown) in addition to other members of the TGFβ signaling family may be sensitive indicators of ESC differentiation. Oct3/4, Sox-2 and nanog in contrast while expressed by ES cells may not be as good in assessing ES cell to embryoid body differentiation. While Oct-3/4 expression declined markedly in both BG02 EBs and pooled ES derived EBs compared to ES the decline was slow and not as rapid as that of other genes. This may be because Oct3/4 expression persists in germ cells that are derived from ESC [31]. Likewise our analysis of nanog showed variable results and there was a discrepancy between the microarray and PCR results and we noted an increase in nanog levels in Day 21 BG02-EB samples by microarray. The reason for this difference is not clear. It is possible that microarray is detecting one of the eleven processed pseudogenes for nanog and that its expression on day 21 represents crosshybridization. Alternatively, nanog expression may be increased as a result of differentiation. This conclusion is supported by a recent report that nanog is expressed in mature tissues [32]. We have confirmed that nanog expression is indeed present in some germ cells and early neuronal populations (data not shown). These tests were performed by RT-PCR using multiple primers and double label immunocytochemistry (results not shown). Future studies will examine expression of nanog in EBs using specific antibody and its function in differentiated cells. At present we recommend that nanog expression should be confirmed by PCR or immunocytochemistry and it should not be included as an initial indicator of the ES cell state and differentiation.

In addition to confirming the downregulation of hESC specific genes our study identified a set 333 genes that were uniquely overexpressed at ≥ 3 fold in day 13 BG02 EB compared to undifferentiated BG02 ES cell lines. Out of these 333 genes 194 genes also showed ≥ 3 fold over expression in EB samples prepared from WiCell lines. This significant similarity of unique genes in BG02 and WiCell line derived EBs suggest that this subset of genes can be classified as EB specific (induced as ESC differentiate) and that embryoid bodies derived from different ES cells maintain significant similarity with each other. These EB specific genes like the ES cell specific genes may be useful in distinguishing EBs from ES cells.

A detailed analysis of upregulated genes in EB indicated that a) differentiation genes, b) cell signaling, cellular process and cell cycle genes, c) cytoskeleton or cell motility genes and d) metabolism and DNA and RNA related genes were modulated. In addition, 27 hypothetical genes, 8 unknown genes and 2 zinc finger genes were upregulated in BG02 derived EB and WiCell lines derived EB. The status of upregulated genes identified by microarray studies was confirmed by MPSS analysis. Among 194 genes, 148 genes showed higher expression in EB by MPSS analysis. Overexpression of 33 genes in EBs compared to HuURNA was confirmed using alternate large-scale array. The expression of a subset of the remaining 13 genes was confirmed by RT-PCR analysis. These results suggest that microarray analysis is a useful complement to MPSS and may identify a overlapping but non-identical set of genes. Among the genes that were upregulated in both EB samples is HAND1 (heart and neural crest derivatives 1). HAND1 was dramatically up regulated (40 fold compared to 6 fold in undifferentiated ES cells). HAND1 belongs to basic helix-loop-helix family of transcription factor, may be required for early trophoblast differentiation as well as the differentiation of heart tissue [33].

Additionally we noted that several markers of early differentiation such as keratin, actin and beta tubulin, which are present at low levels in ESC, were dramatically upregulated as EBs differentiated. We suggest that these markers can serve as sensitive indicators of the process of differentiation and when coupled with the down regulation of ESC markers may reliably distinguish the state of ES cell cultures. Future experiments will assess the sensitivity of a combination of markers identified in this study to temporally profile the process of differentiation. A recent study (34) has compared gene expression of 24-marker genes between EBs differentiated with eight different growth factors and hESC. Similar to our observation by microarray analysis, this study showed over expression of genes related to ectoderm differentiation such as Keratin-8, Keratin-18 and Keratin-19 when differentiated by retinoic acid. A highly significant upregulation of mesodermal related genes e.g., HAND1 was also observed which confirms our observation.

Our results further show that many genes that are induced or upregulated as EBs differentiate remain elevated at Day 21 of differentiation. These genes represent good markers for distinguishing differentiated cells from ESC. In addition we noted that several genes showed differential expression when levels of expression in Day 13 and Day 21 BG02-EB's was compared. Thirty-three of the 194 genes upregulated at Day 13 were down modulated at day21 EB. Other genes such as NPHP3, CAPN1, and CNTN6 were up regulated in day 21 BG02-EB's when compared to day 13 EB. These later appearing genes may reflect additional derivatives that appear as EBs undergo further differentiation. These later appearing markers along with markers that are down regulated can be used to distinguish early from late EBs.

A recent study has characterized differences in gene expression between mouse and human ESC (35). This study identified differences between mouse and human ESC and reported that differences were species specific rather than arising from differing culture conditions. In our current study, while the expression of many genes was similar in rodent and human cells significant differences were found. For example the expression of vimentin, eomesodermin, SSEA4, AFP, IL6ST and HEB was found in hESC but not in mouse ES cells.

The large oligonucleotide arrays have allowed us to build a comprehensive data set that includes hundreds of genes (both novel and unknown) that are either upregulated or downregulated as hESC differentiate. The list of genes has been validated by testing on different cells lines, testing using different oligonucleotide sequences to different regions of the genes and testing arrays used in different formats. Our results show clearly that while small differences exist as different techniques are used the core set of markers is quite robust, the differences in expression quite large and are readily detectable even using a relatively insensitive method such as a microarray. Our results show further that restricting the analysis to only this set of genes as would be necessary in focused array does not reduce the sensitivity of the assessment. Indeed, based on our results we would suggest that an order of magnitude fewer genes may be sufficient if they are appropriately selected from the lists. We have recently developed a focused array that includes genes that are down regulated as hESC differentiate as well as those that are induced as cells differentiate [36].

## Conclusion

In conclusion, a similar pattern of gene expression profile was observed in two differentiated embryoid body samples derived from different embryonic stem cell lines. A consistent and marked downmodulation of most of the "stemness" genes was observed in embryoid bodies indicating that relative levels of these genes can be used to assess the transition of ES cells to embryoid bodies. In addition, we show that 194 unique genes are over expressed in EB and that this subset serves as a complement to the previously characterized "stemness genes" in assessing the degree of differentiation of ES lines and differentiating embryoid bodies. Further validation and confirmation of these data with MPSS and RT-PCR documents the usefulness of high throughput microarray technology and identifies an additional set of previously unknown genes that are likely important in regulating the process of differentiation. These EB enriched genes in combination with downmodulated stemness genes may serve as biomarkers to monitor the transition of undifferentiated human embryonic stem cells to differentiated embryoid bodies.

## Methods

### Isolation and growth of ES cells and differentiation into EB

BG02 ESC were derived at Bresagen Inc., Athens, GA (37) and pooled samples (WiCell lines: WA01, WA07 and WA09, also termed as GE01, GE07 and GE09 respectively) were derived at Wisconsin Alumni Research Foundation, Wicell Research Institute, Madison, WI. (38). ESC were maintained on inactivated mouse embryonic fibroblasts (MEF) feeder cells in Dulbecco's Modified Eagle's Medium (DMEM) supplemented with 15% fetal bovine serum (FBS), 5% knockout serum replacement (KSR), 2 mM non-essential amino acids, 2 mM L-glutamine, 50 μg/ml Penn-Strep (all from Invitrogen, Corporation, Carlsbad, CA), 0.1 mM β-mercaptoethanol (Specialty Media), and 4 ng/ml of basic fibroblast growth factor (bFGF, Sigma). These cell lines were maintained as previously described [3,7,39]. Cells were passaged by incubation in cell dissociation buffer (Invitrogen), dissociated, and then seeded at about 20000 cells/cm^2^. hESC harvested for studies were found to be free of MEF as previously described [30,35].

Embryoid body outgrowths (hEB) were prepared from BG02 cells and pooled samples of ESC (pooled EB) as described [23,30]. Briefly, confluent plates of undifferentiated hESC were used to generate hEBs by a brief exposure to collagenase IV and small clusters of cells were obtained by scraping with a pipette. Cell clusters were resuspended in differentiation medium (KO-DMEM supplemented with glutamine, NEAA, and BME as described for the undifferentiated ESC [8], with 20% FBS in place of 20% serum replacement (SR) and no preconditioning by MEFs) and transferred to individual wells of low adhesion 6-well plates (Costar). After 4 days in suspension, cells were transferred to tissue culture 6-well plates pre-coated with gelatin. Cells were harvested for the preparation of total RNA from BG02 EB on day 13 and day 21 and from pooled EB on day 14. Based on morphological evaluation undifferentiated hESC were also identified in certain regions of differentiating colonies even after 21 days of *in vitro *culture, suggesting that hESC can undergo several cell divisions in "differentiation promoting" culture conditions while maintaining their pluripotent phenotype [40].

### Microarray studies

High quality oligonucleotide glass arrays were produced containing a total of 16,659 seventy-mer oligonucleotides chosen from 750 bases of the 3' end of each ORF (Operon Inc. Valencia, CA). The array includes probes for 2121 hypothetical proteins and 18-expressed sequence tags (ESTs) and spans approximately 50% of the human genome (Operon Inc., Valencia, CA). The arrays were produced in house by spotting oligonucleotides on poly-L-lysine coated glass slides by Gene Machines robotics (Omnigrid, San Carlos, CA). We have followed the MIAME (minimum information about a microarray experiment) guidelines for the presentation of our data [41].

#### i) Probe preparation

Total RNA was isolated from hESC lines derived pellet by using Trizol reagent (Invitrogen). Total human universal RNA (huURNA) isolated from a collection of adult human tissues to represent a broad range of expressed genes from both male and female donors (BD Biosciences, Palo Alto, CA) served as a universal reference control in the competitive hybridization. Labeled cDNA probes were produced as described [42]. Briefly, 5 μg of total RNA was dissolved in 12 μl of DEPC water and incubated at 70°C for 5 minutes along with 1 μl of aminoallyl-oligo dT (5' amino-modified) primer and quickly chilled for 3 minutes. Then, 2 μl 10× first strand buffer, 1.5 μl SSII enzyme (Stratagene, La Jolla, CA), 1.5 μl 20× aminoallyl dUTP and 2 μl of 0.1 M DTT were added and incubated for 90 minutes at 42°C. After incubation, volume of the reaction mixture was raised to 60 μl with 40 μl of DEPC water.

cDNA was purified by MinElute column (Qiagen, Valencia, CA). 300 μl of Binding buffer PB was added to the coupled cDNA, and the mixture applied to the MinElute column, and centrifuged for 1 min at max speed. After discharging the flow-through, 600 μl of washing buffer PE was added to the column, and centrifuged for 1 min at max speed. The flow-through was discharged and the washing repeated. Then the columns were placed into a fresh eppendorf tube and 15 μl elution buffer added to the center of the membrane, incubated for 1 min at room temperature, centrifuged for 1 min at max speed and probe collected. The probe was dried in speed-vac for 16 minutes. Finally, 5 μl of 2× coupling buffer and 5 μl Cy3 and Cy5 dye mixed into the control (huURNA) and experimental cDNAs (huES cell-derived) and incubated at room temperature in dark for 90 minutes. After incubation, the volume was raised to 60 μl by 50 μl DEPC water and then cDNA was purified by MinElute column and eluted with 13 μl elution buffer by centrifugation.

#### ii) Hybridization

For hybridization, 36 μl hybridization mixture [26 μl cDNA mixture, 1 μl (10 μg) COT-1 DNA, 1 μl (8–10 μg) poly(dA), 1 μl yeast tRNA (4 μg), 6 μl 20× SSC and 1 μl 10% SDS] was pre-heated at 100°C for 2 minutes and cooled for 1 minute. Total volume of probe was added on the array and covered with cover slip (22 mm × 40 mm). Slides are placed in hybridization chamber and 20 μl water was added to far end of slide (to maintain humidity), and incubated overnight (10–16 hours) at 65°C. Slides were then washed for 2 minutes each into 2× SSC, 1× SSC and 0.1× SSC and spin-dried.

#### iii) Data filtration, normalization, and analysis

Microarray slides were scanned in both Cy3 (532 nm) and Cy5 (635 nm) channels using Axon GenePix 4000B scanner (Axon Instruments, Inc., Foster City, CA) with a 10-micron resolution. Scanned microarray images were exported as TIFF files to GenePix Pro 3.0 software for image analysis. The raw images were collected at 16-bit/pixel resolutions with 0 to 65,535 count dynamic range. The area surrounding each spot image was used to calculate a local background and subtracted from each spot before Cy5:Cy3 ratio calculation. The average of the resulting total Cy3 and Cy5 signal gave a ratio that was used to normalize the signals. Each microarray experiment was globally normalized to make the median value of the log2-ratio equal to zero. The normalization process corrects for dye bias, PMT (Photo multiplier tube) voltage imbalance, and variations between channels in the amounts of the labeled cDNA probes hybridized. The data files representing the differentially expressed genes were then created.

For advanced data analysis, data files (in gpr format) and image (in jpeg format) were imported into mAdb (microarray database), and analyzed by software tools provided by National Institutes Health Center for Information Technology. Spots with confidence interval of 99 (>3 fold) with at least 150-fluorescence intensity in either channel or 30 μm spot size were considered as good quality spots for analysis. These advanced filters prevented the potential effect of the poor quality spots in data analysis.

### RT-PCR analysis

Total RNA was isolated from both ES and EB cell pellets using the RNeasy Qiagen mini protocol and kit (Qiagen, Valencia, CA). cDNA was synthesized using 1 μg of total RNA in a 20 μl reaction. Superscript II (Invitrogen, Carlsbad, CA) and oligo (dT)_12–18 _primers were used according to the manufacturer's instructions (Invitrogen). The list of primers used for RT-PCR was described in supplemental table 6S (see additional file 8) and annealing conditions are described previously [35].

### MPSS analysis and EST enumeration

We compared microarray results with published MPSS and EST enumeration databases as previously described [30,23]. In brief, MPSS was performed using RNA samples from feeder free cultures of differentiated (day 14 pooled EB derived from WiCell lines) and undifferentiated (passage 35–45) WA01, WA07, and WA09 lines (pooled ESC: pooled samples of ESC). The mRNA was converted to cDNA and digested with DpnII. The last DpnII site and the downstream 16 bases were cloned into Megaclone vectors and their sequences (signatures) were determined according to the MPSS protocol [43,44]. A total of 2,403,315 and 2,591,008 sequences were read for pooled ESC and pooled EB respectively, and 36,498 and 26,599 unique signatures were identified. The abundance for each signature was converted to transcripts per million (tpm) for the purpose of comparison between samples. Signatures at an abundance of less than 4 tpm in at least one of samples or those that were not detected in at least two runs across multiple samples were removed and a total of 24, 229 (pooled ES) and 17,970 (pooled EB) sequences were analyzed further.

EST frequency counts of genes expressed in EB samples were performed as described [23]. Briefly, cDNA libraries of hESC lines (WiCell WA01, WA07, and WA09) grown in feeder free conditions and that of EB's derived from the same cell lines were constructed and submitted for EST sequencing. The EST sequences were assembled into overlapping sequence assemblies and mapped to the UniGene database of non-redundant human transcripts. Expression levels were assessed by counting the number of ESTs for a particular gene that was derived from the undifferentiated hESC and comparing them to the number of ESTs derived from the EB samples. Statistical significance was determined using the Fisher Exact Test [45] using a p-value of p≤ 0.05.

### MPSS signature classification and annotation

To generate a complete, annotated human signature database, we extracted all the possible signatures ("*virtual signatures*") from the human genome sequence (hg16, July 2003, Golden Path, UCSC) and the human Unigene sequences (Unigene build #163). The extracted virtual signatures were classified and ranked according to their positions and the presence of polyA tail and polyA signal features in the source sequence as described [46].

### Immunocytochemistry

Neural cells derived from hESC were processed for immunocytochemistry as described previously [47]. The human Oct3/4 (1:1000) and Sox2 (1:1000) antibodies were obtained form R&D Systems. Bis-benzamide (1:1000, Sigma) was used to identify the nuclei. The secondary antibodies anti-mouse IgG 568 (1:500) and anti-goat IgG 568 (1:500) were purchased from Molecular Probes. Images were captured on an Olympus fluorescence microscope.

## List of abbreviations

hESC-Human embryonic stem cells, EB-embryoid bodies, hEB-Human embryoid bodies, PES-Pooled ES or Wicell derived ES, PEB-Pooled EB, huURNA-Human Universal Reference RNA, MPSS-massively parallel signature sequencing

## Authors' contributions

Authors from FDA/CBER conceived the idea of examining gene expression between ESC and EB cells, performed all microarray hybridizations, performed data analysis, interpreted results, drafted the manuscript and negotiated for acceptance and final publication. Authors from NIH/NIA helped design the study, performed RT-PCR and IHC analyses. NIH/NIDA authors performed RT-PCR analysis to confirm expression of some of the genes. Authors from Bresagen Inc. provided stem cell lines to NIA authors and RNA was extracted at NIA. All authors have read and approved the final manuscript.

## Supplementary Material

Additional File 1 Jpeg files of all five representative images from five different ES and EB samples (cy5 labeled) hybridized against Human Universal Reference RNA (HuURNA) (cy3 labeled). BG02-ES. Click here for file

Additional File 2 Jpeg files of all five representative images from five different ES and EB samples (cy5 labeled) hybridized against Human Universal Reference RNA (HuURNA) (cy3 labeled). Day21-BG02-EB. Click here for file

Additional File 3 Jpeg files of all five representative images from five different ES and EB samples (cy5 labeled) hybridized against Human Universal Reference RNA (HuURNA) (cy3 labeled). Day13-BG02-EB. Click here for file

Additional File 4 Jpeg files of all five representative images from five different ES and EB samples (cy5 labeled) hybridized against Human Universal Reference RNA (HuURNA) (cy3 labeled). Pooled-EB. Click here for file

Additional File 5 Jpeg files of all five representative images from five different ES and EB samples (cy5 labeled) hybridized against Human Universal Reference RNA (HuURNA) (cy3 labeled). Pooled-ES. Click here for file

Additional File 6 Supplementary file -1Sa that represents excel file representation of ≥ 2 fold over expressed genes in BG02-ESC samples hybridized against HuURNA Click here for file

Additional File 7 Supplementary file -1Sb that represents excel file representation of ≥ 2 fold over expressed genes in Pooled-ESC samples hybridized against HuURNA Click here for file

Additional File 8 Represents Supplementary table 2S, 3S, 4S, 5S and 6S in word format with heading and legends mentioned separately in each table Click here for file

Additional File 9 Represents the tables 1–10 in word format with heading and legends mentioned separately in each table Click here for file
