# Supplementary figures and images for: Comparison of the gene expression profile of undifferentiated human embryonic stem cell lines and differentiating embryoid bodies
Source: BMC Dev Biol. 2005 Oct 5;5:22. doi: 10.1186/1471-213X-5-22 (PMC1260016; doi:10.1186/1471-213X-5-22)

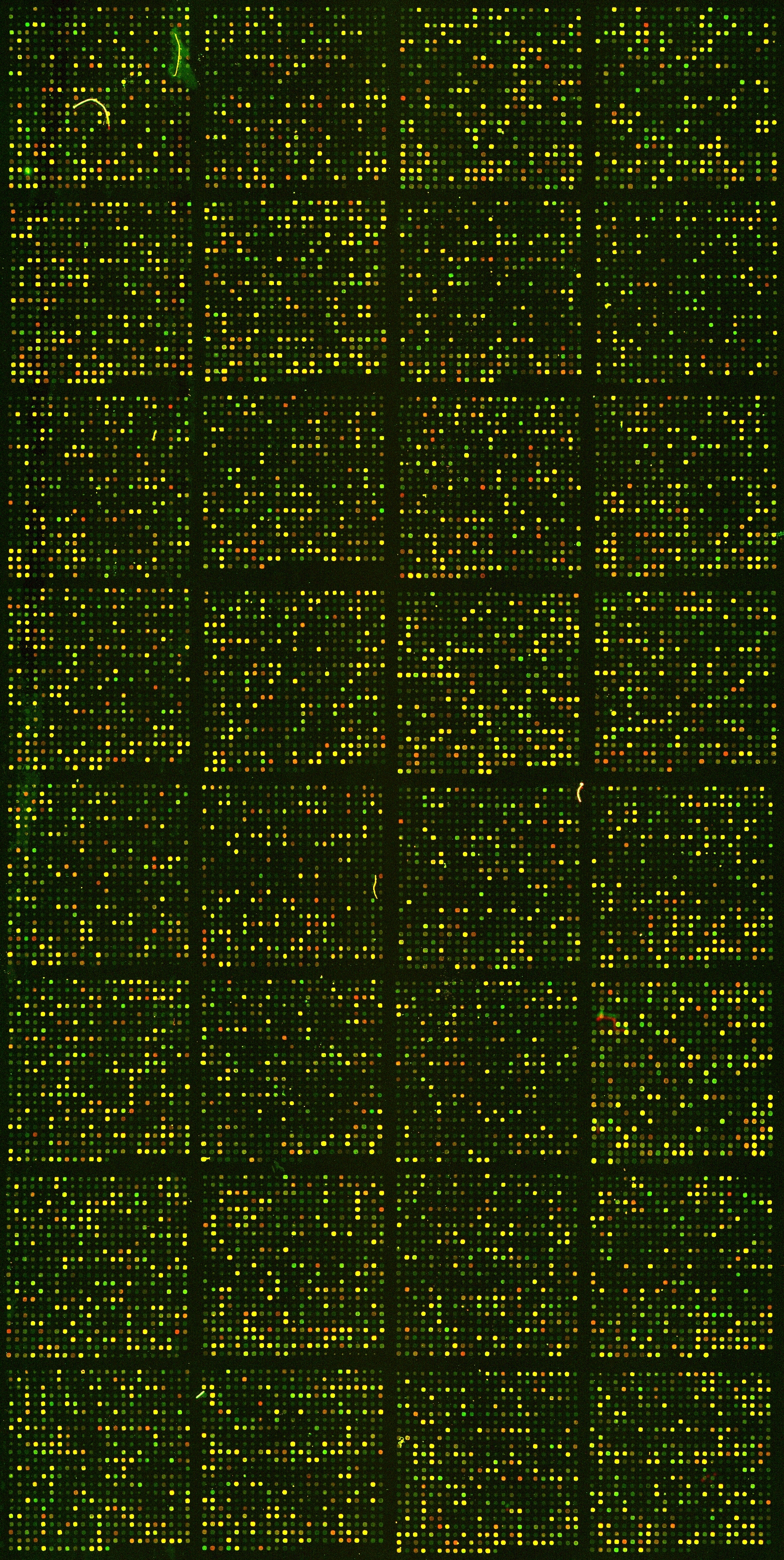

Supplement: Additional File 1 — Jpeg files of all five representative images from five different ES and EB samples (cy5 labeled) hybridized against Human Universal Reference RNA (HuURNA) (cy3 labeled). BG02-ES. [file 1471-213X-5-22-S1.jpeg]

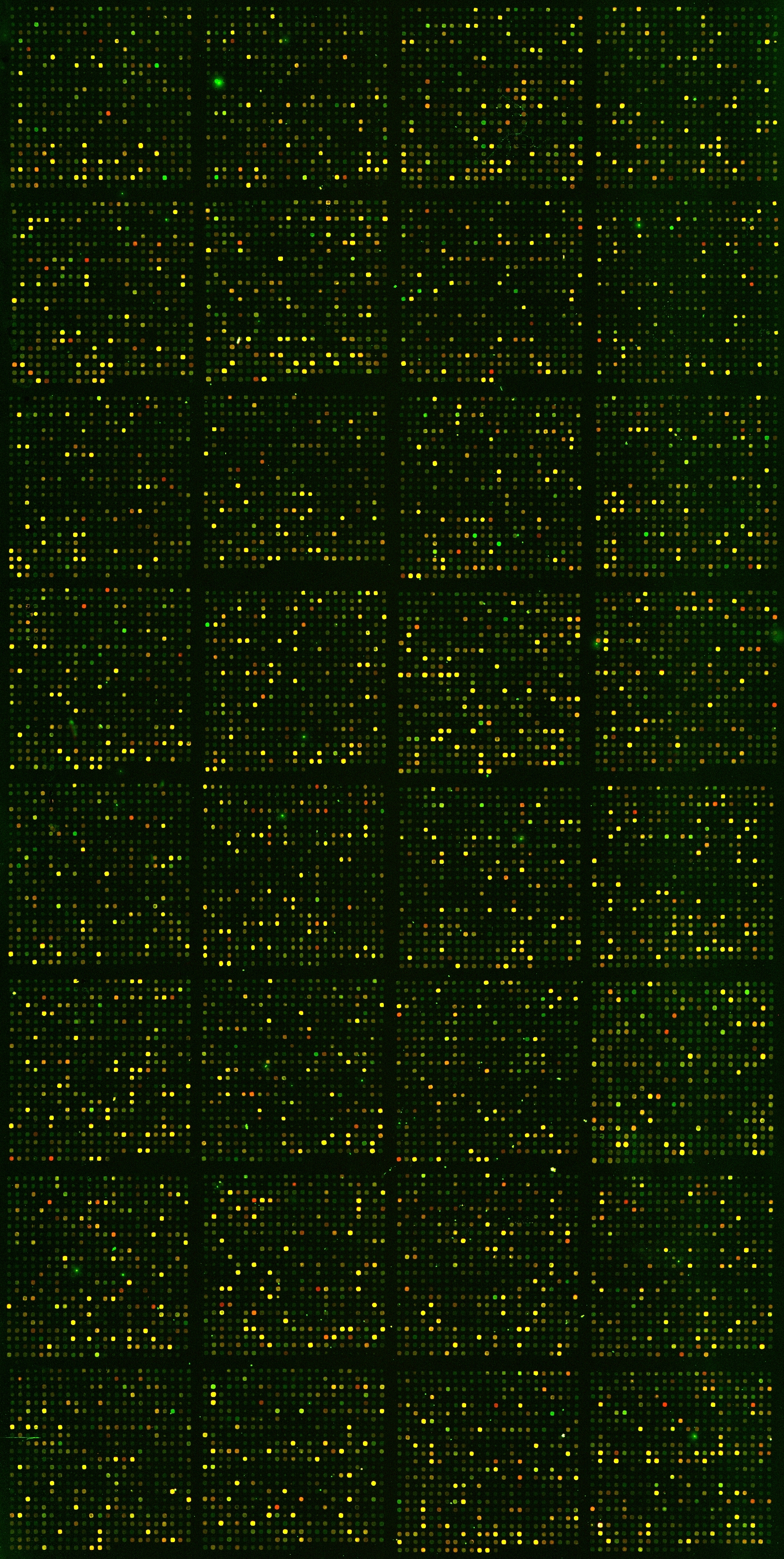

Supplement: Additional File 2 — Jpeg files of all five representative images from five different ES and EB samples (cy5 labeled) hybridized against Human Universal Reference RNA (HuURNA) (cy3 labeled). Day21-BG02-EB. [file 1471-213X-5-22-S2.jpeg]

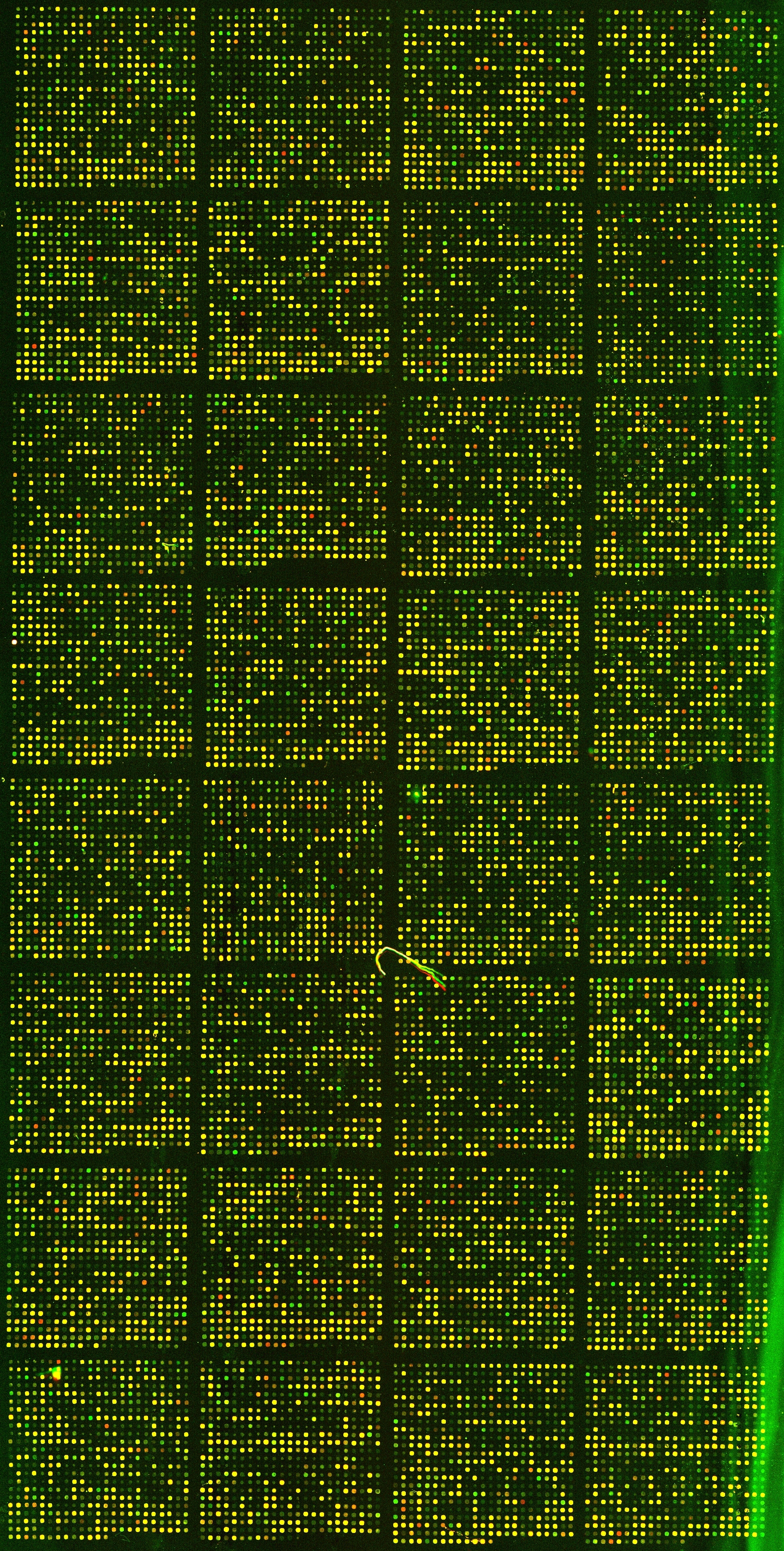

Supplement: Additional File 3 — Jpeg files of all five representative images from five different ES and EB samples (cy5 labeled) hybridized against Human Universal Reference RNA (HuURNA) (cy3 labeled). Day13-BG02-EB. [file 1471-213X-5-22-S3.jpeg]

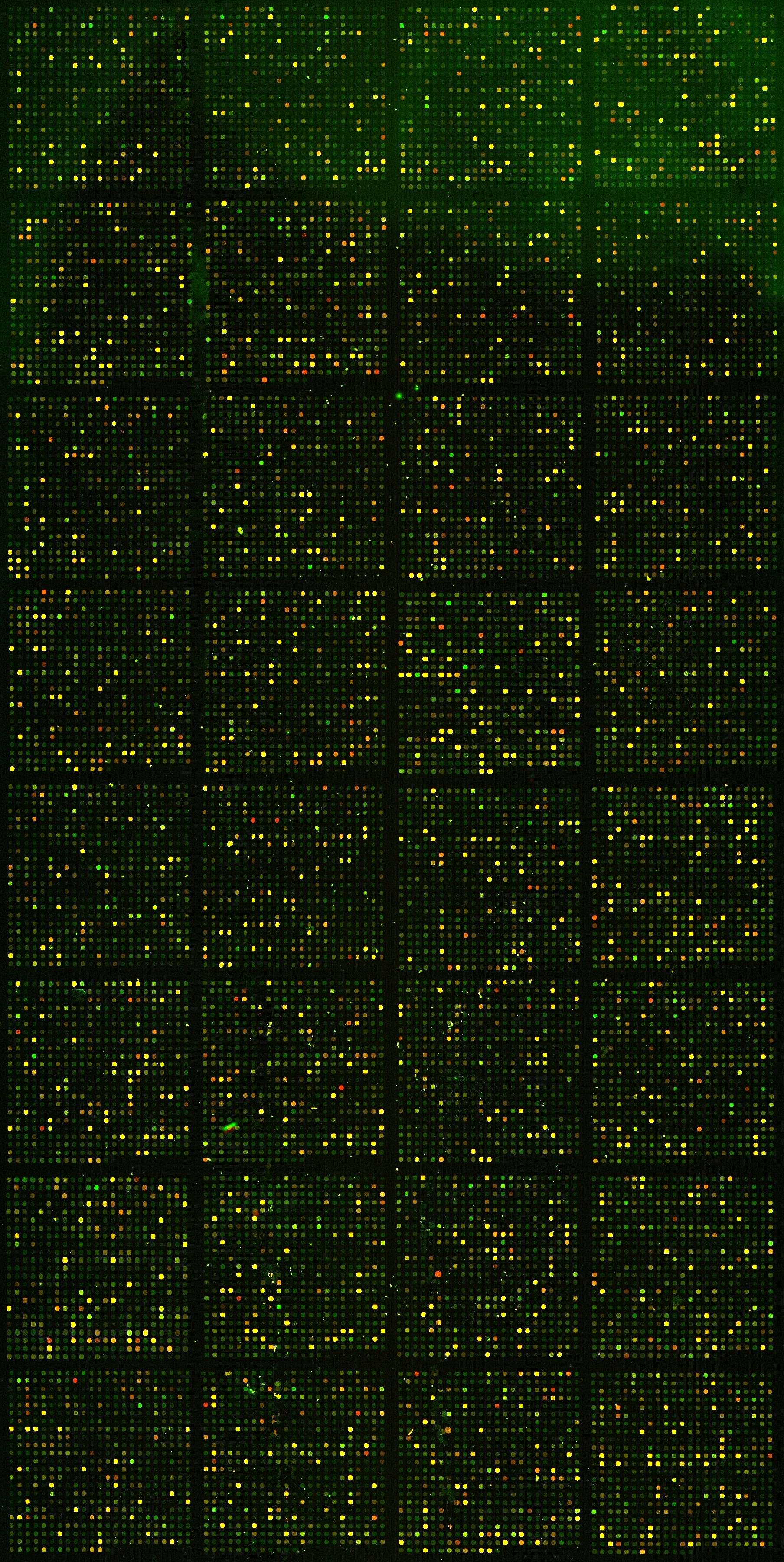

Supplement: Additional File 4 — Jpeg files of all five representative images from five different ES and EB samples (cy5 labeled) hybridized against Human Universal Reference RNA (HuURNA) (cy3 labeled). Pooled-EB. [file 1471-213X-5-22-S4.jpeg]

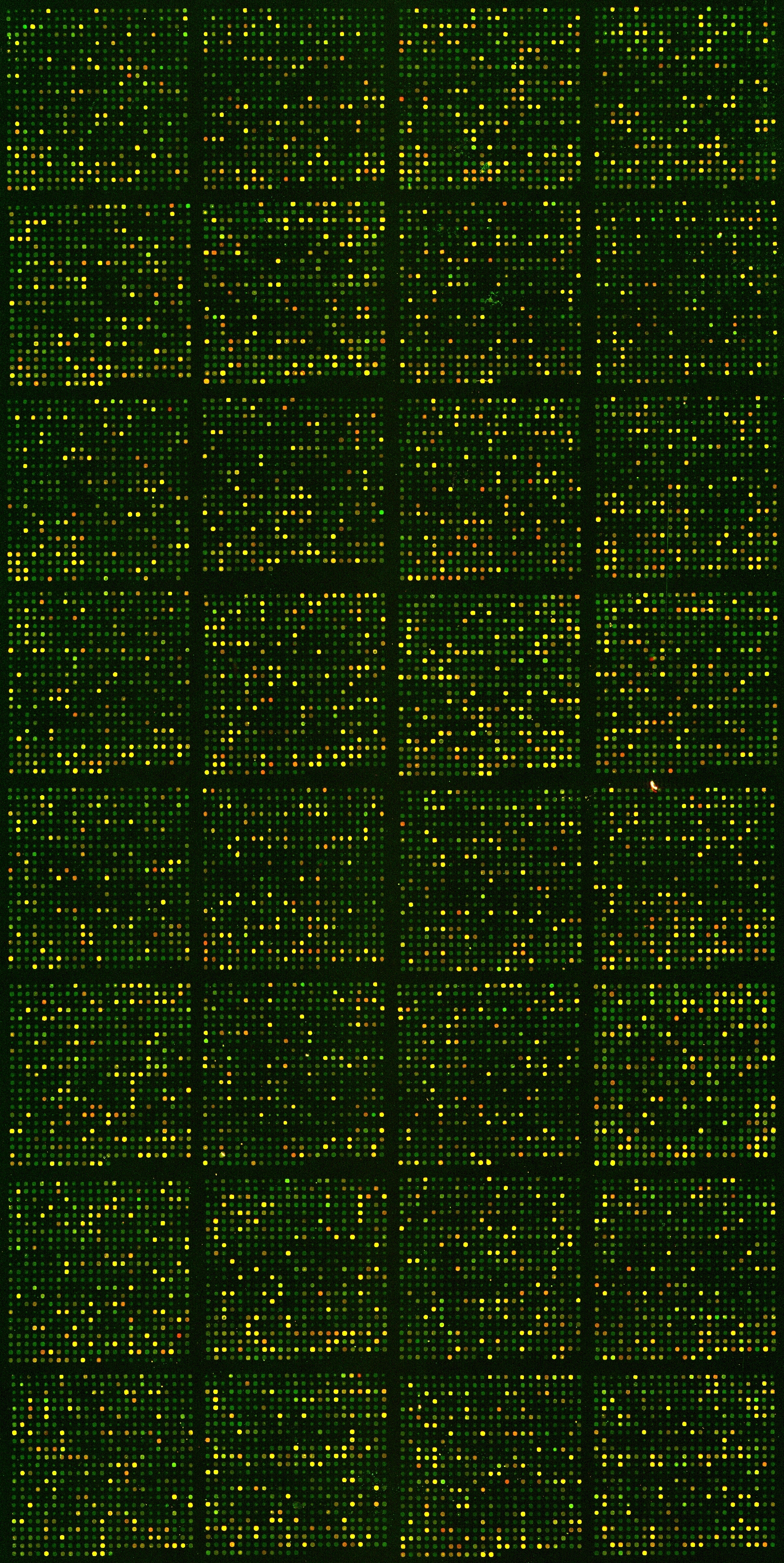

Supplement: Additional File 5 — Jpeg files of all five representative images from five different ES and EB samples (cy5 labeled) hybridized against Human Universal Reference RNA (HuURNA) (cy3 labeled). Pooled-ES. [file 1471-213X-5-22-S5.jpeg]
