# Supplementary material for: Comparison of the gene expression profile of undifferentiated human embryonic stem cell lines and differentiating embryoid bodies
Source: BMC Dev Biol. 2005 Oct 5;5:22. doi: 10.1186/1471-213X-5-22 (PMC1260016; doi:10.1186/1471-213X-5-22)
Supplement: Additional File 8 — Represents Supplementary table 2S, 3S, 4S, 5S and 6S in word format with heading and legends mentioned separately in each table [file 1471-213X-5-22-S8.doc]

# Supplementary table-2: Genes up-regulated in both ES and EB samples in comparison to HuURNA

| RNA-Samples | Total number of genes with ≥ 2 fold over expression than HuURNA |
| --- | --- |
| BG02-**ES** | 2843 |
| Day 13 BG02- EB | 2465 |
| Day 21 BG02-EB | 2716 |
| Pooled **ES** | 2471 |
| Pooled EB | 3053 |

Supplementary table 2: indicates the number of genes expressed in respective ES and EB samples while hybridizing against Human Universal Reference RNA as control

Supplementary Table 3: Categorization of genes expressed uniquely in Day 13 EB at ≥ 3 folds but not observed in BG02-ES

| Category | Gene |
| --- | --- |
| ES cell differentiation markers  (13) | ACIN1, DPPA4, PLP1, COL5A2, COL1A2, COL4A2, COL6A2, COL6A3, P66ALPHA, PABPN1, S100A13, TMSB4X, TWSG1 |
| Cell signaling, Cell growth, Cellular process, Cell cycle (114) | ANAPC13, ARID4B, ATOX1, ARF6, ATP5F1, BIRC7, BMS1L, BST2, CALR, CBX1, CCND2, CCR4, CDH3, CNN2, CRI1, CD164, CD164L1, CD63, CD99, CDC42, CDK4, CDKN1C, CGA, CKLFS6, CNN3, COPZ1, CSE1L, DDX43, ENAH, ERBB2, EFNB1, FAD104, GNAS, GPR48, GTF2A1, Glypican-3, G22P1, GPR43, GRIM19, GSPT1, HVEP3, HYPC, IMP-4, ITM2C, IGFBP2, IL11, IL26, ILF2, INADL, ITPK1, JTB, JUP, KCNA5, KLK14, KCTD10, KDELR1, LRP5, LRP6, LY6E, MAGEA2E, MCF2L, MAGEH1, MFAP4, MGP, MYBL2, M6PRBP1, MAB21L2, MFGE8, NAALD2, NID2, NME4, NEO1, NMI, OPNIMW, PRKAG1, PRKWNK3, PSG9, PDCD4, POSTN, RAB1A, RAB23, RAB27A, RHOBTB1, ROS1, RRBP1, SCRN1, SEC231P, SEC61A1, SERPIN9, SIAHBP1, SEC22L1, SLC17A6, SLC25A3, SLC40A1, SLC9A3R1, SMARCD1, SMCIL1, SRP72, SSR4, SYBL1, TAC3, TAX1BP1, TGFB1, TMEM14C, TP5313, TRIF, TIMM17B, TRIM34, VPS33A, VCIP135, VAT1, VAPA, VAMP3, YWHAE |
| Cytoskeleton or cell motility (9) | ARPC1B, CFL1, GYPC, SPON2, SPTA1, MFAP4, TNNI1, TUBA4, VIM |
| Metabolic activity  DNA and RNA related (24) | ATF5, CDC34, FLJ23233, GABARAP, GLE1L, GNB2L1, GNB3, HNRPH3, KIAA0146, PRPF8, RPL15, RPL17, RPL18, RPL28, RPL30, RPL36, RPLP2, RPS11, RPS16, RPS17, RPS2, RPS26, RPS3, ZC3HAV1 |
| Metabolism (119) | ACLY, ALG5, ANXA6, APG4B, ARHGAP9, ATOX1, ATP5G1, APOA1, ARF6, BG1, CAMK2G, CNOT2, COX6B, COX7C, C5ORF14, C5ORF15, C11ORF23, C11ORF9, C13ORF12, C14ORF47, C14ORF58, C14ORF73, C20ORF59, C6ORF157, C9ORF83, CLSTN2, COBLL1, CTAG2, CXX1, CHST2, DIO3, DPH2L1, DHX40, DRG1, DSCR4, DXYS155E, D1S155E, DDOST, DOK4, EIF4EBP1, ENTPD4, FAM36A, FAM3A, FGB, FBXL14, GALNT10, GBA, GLB1, GOLGA2L, GOLGA6, GORASP2, GARP, GNB3, GLB1, GTF2A1, H3F3B, HEMGN2, HECTD2, H2AFY2, HEPH, HOXB4, HSPC051, INPP5F, LEPREL2, LOC55971, MCM8, MMP2, MRC2, MEIS2, MIF, MRC2, NROB1, NRBP, NME4, PDE3B, PDHA1, PFKFB2, PLAT, PLEKHJ1, PMS2L9, POP1, PPIA. PRDX2, PRKDC, PRR3, PRTFDC1, P4HB, PHGDH, PITX1, PKM2, PLTP, POLR2L, PPP1CA, PPP4R2, PRP19, RAB11F1P1, RNF19, Renin, S100A11, S100A14, STARD3, STK36, SORD, TCF15, TRIP, TTC3, TXNDC7, UBA52, UBE2E3, UBE1, UQCR, USP33, UXT, VPS33A, VNN3, VTN, WFDC3, XYLT2, ZCWCC2 |
| Others (54) | TZFP, ZFHX1B, ZNF160, ZNF224, ZNF302, ZNF306, ZNF350 H-plk  38 Hypothetical proteins and 8 unknown proteins |

Supplementary Table 3: Numbers in parenthesis represent the number of genes under each category. Genes expressed at a 99% confidence interval (≥ 3 fold) are shown

Supplementary table 4: Genes that showed higher expression in Day 13 and Day 21 EB compared to BG02-ES^a^

| Gene | BG02-ES | Day 13 EB | Day 21 EB | Gene | BG02-ES | Day 13 EB | Day 21 EB |
| --- | --- | --- | --- | --- | --- | --- | --- |
| ES differentiation markers |  |  |  | **Cell cycle regulation** |  |  |  |
| *HAND1* | 7 | 41 | - | *BIRC7* | - | 4 | 3 |
| *KRT19* | 8 | 16 | - | CNTN6 | - | - | 3 |
| *Fibronectin1* | 5 | 14 | 3 |  |  |  |  |
| *Profilin1* | 7 | 9 | 7 | **Metabolism** |  |  |  |
| *ENO1* | 7 | 9 | - | NUCKS | 5 | 5 | 8 |
| *TMSB4X* | 4 | 7 | 7 | COX7C | 2 | 3 | 5 |
| *Vimentin* | 2 | 3 | 3 | *MAPK1* | - | 2 | 2 |
| *PLP2* | 2 | 3 | 3 | *MAPK8* | - | 2 | 2 |
| *AFP* | - | 3 | - | *MARCKS* | - | 2 | 2 |
| *KRT10* | - | 2 | - | *CEBPA* | - | 2 | 3 |
| *KRT17* | - | - | 2 | *NFKBIA* | - | - | 3 |
| *Involucrin/IVL* | - | - | 2 | *MAP2K7* | - | - | 2 |
| *KRT-20* | - | - | 2 |  |  |  |  |
| *TUBA3* | 3 | 3 | 4 |  |  |  |  |
| Cytoskeleton or cell motility |  |  |  |  |  |  |  |
| *COL1A2* | - | 27 | 3 |  |  |  |  |
| *COL5A2* | 2 | 7 | - |  |  |  |  |
| *COL4A2* | - | 4 | - |  |  |  |  |
| *NID2* | - | 3 | 3 |  |  |  |  |
| NPHP3 | - | - | 3 |  |  |  |  |
| *CAPN1* | - | - | 3 |  |  |  |  |
| *ZAK* | - | 2 | 6 |  |  |  |  |

###### Supplementary Table-4: ^a^ Indicates the fold expression compared to HuURNA. (-) Indicates ≤1 fold or not expressed

Supplementary table 5: Genes showed higher expression in pooled EB

compared to pooled ES

| Gene | PES^a^ | PEB^a^ | Gene | PES^a^ | PEB^a^ |
| --- | --- | --- | --- | --- | --- |
| ES differentiation markers |  |  | **Metabolism** |  |  |
| *Fibronectin1* | 2 | 44 | *COX7C* | - | 5 |
| *KRT19* | 1 | 19 | *NUCKS* | 2 | 8 |
| *ENO1* | 2 | 8 | *MARCKS* | 1 | 3 |
| *PLP2* | 1 | 4 | *MAPK1* | 1 | 2 |
| *TUBA3* | 1 | 4 | *MAPK8* | 2 | 4 |
| *Profilin1* | 2 | 6 | *NFKBIA* | - | 2 |
| *TMSB4X* | 2 | 6 |  |  |  |
| *Vimentin* | 1 | 3 | Cytoskeleton or cell motility |  |  |
| *Vimentin* | 1 | 3 | *COL1A2* | - | 10 |
| *HAND1* | 2 | 7 | *COL4A2* | 1 | 6 |
| *Involucrin/IVL* | - | 2 | *NID2* | 2 | 5 |
| *KRT-20* | - | 2 | SPTA1 | - | 3 |
|  |  |  | *CAPN1* | - | 2 |
| **Cell cycle regulation** |  |  | *ZAK* | 1 | 2 |
| *BIRC7* |  |  |  |  |  |
| CNTN6 |  |  |  |  |  |

###### Supple Table-5: ^a^ Indicates the fold expression compared to HuURNA.

###### (-) Indicates ≤ 1 fold or not expressed

Supplementary table 6: Primer sequences used for RT-PCR analysis of some of the EB expressed genes.

**________________________________________________________________________**

Gene Size Primer Sequence (5’ to 3’)

FLJ10134 684 bp FLJ10134-F gcaaaagcgaacctgctatc

FLJ10134-R aacgaggcctgtcagaaaga

FLJ12541 295 bp FLJ12541-F tgttggatgagcttcagtgc

FLJ12541-R cgttgtagagggcagagagg

FLJ22329 819 bp FLJ22329-F acagactcccagttgggttg

FLJ22329-R agggaccacacctgagacac

LOC348262 189 bp LOC348262-F gtggagcgctaggatcagac

LOC348262-R cggtaggggtaggtgtgaga

MDS025 353 bp MDS025-F ctgttggactatgccccact

MDS025-R tccctccttggttgtggtag

MGC35097 152 bp MGC35097-F gctgtctgtgatggtgtgct

MGC35097-R atgctgaggcacagagaggt

C5orf15 445 bp C5orf15 -F gacgacgacgagtctgatga

C5orf15 -R aagcaccaaggcaaaagaga

DDX43 560 bp DDX43 -F aaacgacctatccccaatcc

DDX43 -R gggcgcacatctaacaaaat

FLJ21031 449 bp FLJ21031 -F caaggaggtcgaggctacag

FLJ21031 -R ccacaccccacactcttctt

GBA 450 bp GBA -F atccgcacctacacctatgc

GBA -R gagtaggcggacattgtggt

LOC347544 266 bp LOC347544 -F tactgtgggcaggtgtttga

LOC347544 -R ccctgcccttagaagaaggt

MCM8 315 bp MCM8-F caaaccccacagtttttgct

MCM8-R gcaagccaaggttttctcag

PRO1776 285 bp PRO1776-F agggaggtgagttccttggt

PRO1776-R gcatccaccacaaactgatg

PRO2792 365 bp PRO2792-F gattggggaagatttgctga

PRO2792-R gcttgttttctgcctccttg

RNABP10 250 bp RNABP10 -F tgagagctggacagagcaga

RNABP10 –R ctcaggacgaaggcaggtag

RSHL2 444 bp RSHL2 -F gcgacaccgagaagaaaaag

RSHL2 –R tctaaaagcagctcccgtgt

ZFHX1B 393 bp ZFHX1B-F ttcctgggctacgaccatac

ZFHX1B-R gccttgagtgctcgataagg

ZNF306 678 bp ZNF306-F aggtggtggtgctattggag

ZNF306-R agggtttctcaccagtgtgg

ZNF350 501 bp ZNF350-F gtggcaaagcctttctcaag

ZNF350-R ccttggctgcttcttgtttc

ABCG2 684 bp ABCG2-F gtttatccgtggtgtgtctgg

ABCG2-R ctgagctatagaggcctggg

Connexin 43 294 bp Connexin 43-F taccatgcgaccagtggtgcgct

Connexin 43-R gaattctggttatcatcggggaa

Dppa 5 (Esg1) 353 bp Dppa 5-F atgggaactctcccggcacg

Dppa 5-R tcacttcatccaagggccta

GATA4 577 bp GATA4-F ctccttcaggcagtgagagc

GATA4-R gagatgcagtgtgctcgtgc

hTERT 187 bp hTERT-F agctatgcccggacctccat

hTERT-R gcctgcagcaggaggatctt

Nanog 158 bp Nanog-F caaaggcaaacaacccactt

Nanog-R tctgctggaggctgaggtat

# Nestin 389 bp Nestin-F cagcgttggaacagaggttgg

Nestin-R tggcacaggtgtctcaagggtag

Oct3/4 171 bp Oct3/4-F cttgctgcagaagtgggtggaggaa

Oct3/4-R ctgcagtgtgggtttcgggca

Rex-1 559 bp Rex-1-F tgaaagcccacatcctaacg

Rex-1-R caagctatcctcctgctttgg

Sox-1 425 bp Sox-1-F gcggaaagcgttttctttg

Sox-1-R taatctgacttctcctccc

Sox-2 437 bp Sox-2-F atgcaccgctacgacgtga

Sox-2-R cttttgcacccctcccattt

Utf-1 230 bp Utf-1-F accagctgctgaccttgaac

Utf-1-R ttgaacgtacccaagaacga

APOA1 260 bp APOA1-F catttctggcagcaagatga

APOA1-R aggccctctgtctccttttc

ATOX1 257 bp ATOX1-F aggctgtgctgaagctgtct

ATOX1-R aagtcccaggtctgtctgga

CD164L1 337 bp CD164L1-F aaccagacctcacccatcag

CD164L1-R gttctgttgggctcttgctc

COL1A2 391 bp COL1A2-F gaaaacatcccagccaagaa

COL1A2-R ggcgtgatggcttatttgtt

NAALAD2 547 bp NAALAD2-F ttgccaagaaaatccaaacc

NAALAD2-R gctggatagcctggagtgag

SLC40A1 505 bp SLC40A1-F cccaaggctgttgtgttttt

SLC40A1-R ccatccatggtacatggtca

TFGB1 440 bp TFGB1-F agcagccctaccactctcaa

TFGB1-R gctggatgttgttggtgatg

**G3PDH** 474 bp hGAPD-F gctcagacaccatggggaaggt

hGAPD-R gtggtgcaggaggcattgctga

**Beta Actin** 838 bp hACTB-F caccttctacaatgagctgcg

HACTB-R tgcttgctgatccacatctgc

--------------------------------------------------------------------------------------------------------
