# Supplementary material for: Comparison of the gene expression profile of undifferentiated human embryonic stem cell lines and differentiating embryoid bodies
Source: BMC Dev Biol. 2005 Oct 5;5:22. doi: 10.1186/1471-213X-5-22 (PMC1260016; doi:10.1186/1471-213X-5-22)
Supplement: Additional File 9 — Represents the tables 1–10 in word format with heading and legends mentioned separately in each table [file 1471-213X-5-22-S9.doc]

Table-1: Genes that were down modulated in Pooled samples of EB

BG02-EB derived at day 13^a^

| Gene | BG02-ES | Day 13-BG02- EB | PES | PEB |
| --- | --- | --- | --- | --- |
| **ES cell markers** |  |  |  |  |
| Galanin | 33 | 4 | 5 | 4 |
| POU5F1 | 28 | 3 | 23 | 3 |
| *GTCM-1* | 14 | 12 | 15 | 9 |
| *GDF3* | 13 | 2 | 4 | - |
| *GJA1* | 10 | 4 | 6 | 2 |
| *Nanog* | 6 | -^b^ | 8 | - |
| Cell cycle regulation |  |  |  |  |
| *Lin-28* | 28 | 13 | 13 | 10 |
| *SEMA6A* | 25 | 3 | 5 | 2 |
| *CRABP2* | 19 | 17 | 11 | - |
| *LEFTB* | 18 | - | 24 | - |
| *CCNB1* | 10 | 2 | 6 | 2 |
| *CCNC* | 9 | 3 | 4 | 3 |
| *SFRP2* | 8 | 3 | 7 | 2 |
| *IMP-2* | 8 | 3 | 7 | 5 |
| *TK1* | 7 | 3 | 4 | 3 |
| *SET* | 6 | 2 | 4 | 3 |
| *BRIX* | 6 | 3 | 4 | 3 |
| *CRABP1* | 5 | 3 | 11 | 11 |
| *STK12* | 4 | 3 | 8 | 2 |
| *MAD2L2* | 4 | 2 | 5 | 5 |
| *PTTG1* | 6 | 3 | 3 | 5 |
| *NME2* | 11 | 7 | 3 | 8 |
| *CDC2* | 9 | 3 | 6 | 7 |
| Metabolism **DNA & RNA** |  |  |  |  |
| NPM1 | 23 | 13 | 8 | - |
| SSB | 20 | 6 | 8 | 6 |
| *DDX21* | 14 | - | 5 | 4 |
| *NS* | 9 | 3 | 5 | 4 |
| *Jade-1* | 8 | 2 | 5 | 2 |
| *RAMP* | 6 | - | 4 | - |
| *HMGB2* | 6 | 3 | 5 | 4 |
| *EPRS* | 5 | - | 5 | 2 |
| *RPL7* | 4 | 3 | 4 | 4 |
| SNRPF | 23 | 6 | 3 | 6 |
| *EIF4A1* | 18 | 9 | 4 | 6 |
| *RPL6* | 17 | 8 | 5 | 8 |
| *HMGIY* | 12 | 10 | 9 | 15 |
| *HNRPAB* | 12 | 5 | 3 | 4 |
| *KPNA2* | 11 | 5 | 5 | 6 |
| *NBR2* | 8 | 7 | 4 | 6 |
| *RPL24* | 6 | 3 | 4 | 8 |
| *RPL4* | 6 | 3 | 3 | 10 |
| Metabolism |  |  |  |  |
| *SLC16A1* | 19 | 5 | 10 | 5 |
| *ELOVL6* | 19 | 2 | 12 | 2 |
| *CYP26A1* | 18 | 2 | 14 | 2 |
| *FABP5* | 18 | 4 | 4 | 2 |
| *KIF4A* | 11 | 2 | 4 | - |
| *PSMA2* | 10 | 5 | 4 | 4 |
| *MGST1* | 7 | 2 | 3 | 2 |
| *MTHFD2* | 6 | - | 6 | 2 |
| *MTHFD1* | 5 | 2 | 5 | 2 |
| *HDAC2* | 5 | 3 | 5 | 4 |
| *IDH1* | 5 | - | 4 | - |
| TDGF1 | 5 | - | 7 | 2 |
| *HSSG1* | 5 | - | 3 | - |
| *LAPTM4B* | 3 | - | 4 | - |
| *LDHB* | 15 | 11 | 4 | 9 |
| *SMS* | 14 | 4 | 4 | 5 |
| *PSMA3* | 6 | 4 | 3 | 5 |
| *IMPDH2* | 6 | 5 | 4 | 5 |
| *CCT8* | 12 | - | 4 | 5 |
| Novel genes |  |  |  |  |
| *IFITM1* | 12 | - | 5 | 2 |
| *GSH1* | 12 | 3 | 5 | 2 |
| *PPAT* | 9 | 2 | 6 | - |
| *KIAA1573* | 9 | 2 | 4 | - |
| *C20orf1* | 8 | - | 5 | 4 |
| *Laminin receptor* | 8 | 6 | 4 | 3 |
| *C20orf129* | 5 | - | 5 | 4 |
| *ARL8* | 4 | - | 5 | - |
| *Numatrin* | 17 | 13 | 5 | 16 |
| *C20orf168/ HNRPA1* | 10 | 5 | 5 | 7 |
| *TD-60* | 9 | 4 | 3 | 7 |
| *C15orf15* | 8 | 5 | 4 | 7 |
| Others |  |  |  |  |
| *NASP* | 10 | 2 | 7 | 2 |
| *LRRN1* | 8 | 2 | 8 | 3 |
| Other ES cell markers |  |  |  |  |
| *CER1* | 44 | - | 12 | - |
| *DNMT3B* | 22 | 2 | 34 | - |
| *SOX2* | 4 | 3 | 5 | 3 |

^a^ Numbers represents fold expression compared to HuURNA.

^b^ (-) Indicates not expressed.

77/92 genes showed down modulation in Day 13 BG02 -EB and out of them 53 showed down modulation in PEB. Bold genes showed reverse pattern in PEB (over expression in PES) compared to day 13 BG02 EB.

###### Table-2: Genes that were upregulated in Day 13 EB and Pooled samples of EB

| Gene | BG02-ES | Day 13 BG02-EB | Pooled ES^a^ | Pooled EB^a^ |
| --- | --- | --- | --- | --- |
| *KRT8* | 17 | 45 | 4 | 27 |
| *KRT18* | 23 | 24 | 5 | 23 |
| *TUBB5* | 5 | 8 | 6 | 11 |
| *ACTC* | 22 | 23 | 4 | 6 |
| *RPLP0* | 7 | 8 | 7 | 9 |
| *SERPINH1* | 6 | 8 | 4 | 10 |
| *TUBB4* | 4 | 5 | 4 | 7 |
| *RPS24* | 15 | 17 | 5 | 18 |
| *H-plk /Znf257* | 6 | 7 | 5 | 4 |
| *PITX2* | 7 | 11 | 6 | 4 |

###### Numbers indicate fold expression compared to HuURNA.

###### ^a^PEB and PES were derived from GE01, GE07 and GE09 ES cell lines. Bold genes showed a reverse pattern of expression in PEB compared to day 13 EB

Table-3: Categorization of 194 genes expressed uniquely in Day 13 EB and pooled samples of EB at ≥ 3 folds but not in BG02-ES and pooled samples of ES

| Category^b^ | Gene**^a^** |
| --- | --- |
| ES cell differentiation specific genes  (12) | ACIN1, DPPA4, COL5A2, COL1A2, COL4A2, COL6A2, COL6A3, P66ALPHA, PABPN1, S100A13, TMSB4X, TWSG1 |
| Cellular process (Cell signaling, Cell growth, Cellular process, Cell cycle) (69) | ARID4B, ATOX1, ARF6, BST2, CALR, CCND2, CDKN1C, CNN2, CD164L1, CD99, CDC34, CDC42, CKLFS6, CNN3, COPZ1, DDX43, ERBB2, GNAS, Glypican-3, GPR48, G22P1, GPR43, GSPT1, HIVEP3, HYPC, IMP-4, ITM2C, IGFBP2, IL11, IL26, ILF2, ITPK1, JTB, JUP, KCNA5, KCTD10, KDELR1, LRP5, LRP6, LY6E, MCF2L, MYBL2, MFGE8, NAALD2, NID2, NME4, NEO1, NMI, PRKAG1, PRKWNK3, PSG9, PDCD4, RAB1A, RAB23, RAB27A, RRBP1, SEC61A1, SIAHBP1, SLC40A1, SLC9A3R1, TGFB1, TRIF, TIMM17B, TMEM30A, TRIM34, VCIP135, VAT1, VAPA, YWHAE |
| Cytoskeleton or cell motility (6) | ARPC1B, CFL1, GYPC, SPTA1, TUBA4, VIM |
| Metabolic activity  DNA and RNA related (15) | ATF5, GABARAP, GLE1L, GNB2L1, HNRPH3, , RPL17, RPL18, RPL28, RPL36, RPLP2, RPS11, RPS17, RPS29, RPS3, ZC3HAV1 |
| Metabolism (55) | APG4B, ARHGAP9, APOA1, BG1, COX6B, COX7C, C5ORF15, C11ORF9, C13ORF12, C14ORF47, C14ORF58, C6orf157, COBLL1, CXX1, CHST2, DRG1, DSCR4, DXYS155E, EIF4EBP1, FAM36A, FBXL14, GBA, GOLGA6, H3F3B, H2AFY2, HSPC051, LEPREL2, MCM8, MMP2, MRC2, MIF, NME4, FADS1, PDHA1, PLEKHJ1, PMS2L9, POP1, PPIA. PRDX2, PRKDC, PRR3, PHGDH, PLTP, PPP1CA, RAB11F1P1, S100A11, STARD3, STK36, TTC3, UBA52, UBE2E3, UQCR, VNN3, VTN, XYLT2 |
| Others (37) | ZNF306, ZNF350  26 Hypothetical proteins and 9 unknown proteins |

^a^ indicates unique genes expressed at a 99% confidence interval (≥ 3 fold) in EBs. ^b^ numbers in parenthesis represent the number of genes under each category.

Table-4: Categorization of 37 unknown genes expressed uniquely in EB^a^

| **Gene / Blast symbol** | **Chrom. Location** | **Unigene /**  **Accession**  **No.** | **Predicted protein size** | **Annotation** |
| --- | --- | --- | --- | --- |
| DKFZP761A052 | Xp11.23 | Hs.184029 | 571aa | Hypothetical protein DKFZp761A052 |
| FLJ10134/  DERP7 | 3q12.3 | Hs.104800 | 275aa | Hypothetical protein FLJ10134 / dermal papilla derived protein 7 |
| FLJ12541/  Stra6 | 15q22.33 | Hs.24553 | 667aa | FLJ12541 / stimulated by retinoic acid gene 6 |
| FLJ12650 | 1p35.1 | Hs.436090 | 163aa | Hypothetical protein FLJ1260 |
| FLJ12983 | 7 | AK023045 | 177aa | Hypothetical protein FLJ12983 / LOC340351 |
| FLJ14129 | 8q24.3 | Hs.512740 | 374aa | Hypothetical protein FLJ14129 |
| FLJ21031 | 1p31.3 | AK024684 | 126aa | Hypothetical protein FLJ21031 : Homo sapiens cDNA:clone CAE07336 |
| FLJ22329 | 19p13.12 | Hs.418795 | 622aa | Hypothetical protein FLJ22329 |
| FLJ22761 | 10q22.1 | Hs.445459 | 911aa | Hypothetical protein FLJ22761 |
| FLJ23233 | 19q13.43 | Hs.98593 | 387aa | Hypothetical protein FLJ23233 |
| FLJ14213 | 11p13 | Hs.75307 | 213aa | Hypothetical protein FLJ14213 |
| H1FX | 3q21.3 | Hs.75307 | 213aa | H1 histone family , member X may be related to metabolism |
| KIAA0146 | 2q13 | Hs.381058 | 45aa | KIAA0146 protein (KIAA0146), mRNA |
| KIAA1052 | 11q23.3 | Hs.18624 | 1460aa | KIAA1052 protein |
| KIAA1318/  RGAG1 | Xq23 | Hs.20171 | 1388aa | KIAA1318/ Retrotransposon gag domain containing 1 (RGAG1) |
| KIAA1602 | 2q13 | Hs.143067 | 1189aa | KIAA1602 |
| LOC347544 | Xq28 | XP_293412 | 147aa | Predicted: Homo sapiens similar to ribosomal protein L18a; 60S ribosomal protein L18a (LOC347544) |
| LOC348262 | 17p13.3 | Hs.285165 | 175aa | Hypothetical proteins LOC348262 |
| LOC51215/  PRO0398 | 19 | AF113674.1 | 105aa | Clone FLB1727 / C3: complement component 3 |
| LOC55971 | 7q22.1 | Hs.285165 | 500aa | Insulin receptor tyrosine kinase substrate (LOC55971), mRNA |
| MDS025/  MDS011 | 11q14.1 | Hs.368866 | 254aa | Hypothetical protein MDS025/ Hypothetical protein MDS011 |
| MGC35097 | 3p21.31 | Hs.13781 | 354aa | Hypothetical protein MGC35097 |
| MGC51082 | 19q13.32 | Hs.99093 | 188aa | Hypothetical protein MGC51082 |
| MGC8721/ XTP-3 | 8p12 | Hs.27921 | 339aa | Hypothetical protein MGC8721/ XTP-3 / FLJ22274 |
| PF20 | 2q34 | Hs.6783 | 631aa | PF 20 / WD repeat domain 29 (sperm associated WD repeat proteins) |
| POF1B | Xq21.2 | Hs.267038 | 589aa | Premature ovarian failure 1B (POF1B) |
| PRO1776 | 5 | -^b^ | - | DISCONTINUED in mAdb : Locus ID 55377 was defined by AF119853.1 |
| PRO2792 | 11q23.3 | AF180681 | 1544aa | Hypothetical protein PRO2792 / ARHGEF12 : Rho guanine nucleotide exchange factor (GEF) 12 |
| PRO2852 | 9 | Hs.493756 | 502aa | UBAP1 : Ubiquitin associated protein 1 |
| RANBP10/ RANBP9 | 16q22.1 | Hs.6343 | 620aa | Ran-binding protein 10 / RANBP9 / RANBPM : encodes a protein that binds RAN, a small GTP binding protein belonging to the RAS superfamily that is essential for the translocation of RNA |
| RSHL2 / RSP3 | 6q25.3 | Hs.154628 | 560aa | Radial spokehead-like 2 (RSHL2) : Radial spoke protein 3 or A-kinase anchoring protein (AKAP) involved in neuronal migration during development of CNS |
| UNC13D / UNC13 | 17q25.3 | Hs.41045 | 1090aa | Unc-13 homolog D (C.elegans) may be involved in synaptic transmission |
| ZFHX1B | 2q22 | Hs.34871 | 1214aa | Zinc finger homeobox 1b encodes for SMAD interacting protein 1: considered an important gene for normal embryonic neural crest development. Mutation in this gene causes Hirschsprung's disease. |
| ZC3HAV1 | 7q34 | Hs.133512 | - | zinc finger CCCH type, antiviral 1(ZC3HAV1),transcript variant 2, mRNA. |
| ZNF306 | 6p21.33 | Hs.66774 | 538aa | Zinc finger protein 306 : involved in transcriptional regulation |
| ZNF350 | 19q13.41 | Hs.407694 | 532aa | Zinc finger protein 350 : involved in transcriptional regulation |
| ZP2 | 16p12 | Hs.73982 | 745aa | Zona pellucida glycoptorein 2 (sperm receptor) : may be involved with fertilization and preimplantation development |

^a^Twenty six hypothetical proteins, 2 zinc finger proteins and 9 unknown genes over expressed in EB were analyzed by using bioinformatic tools available at multiple databases, including NCBI (National Center for Biotechnology) blast search, locus link, pubmed, the National Cancer Institute and the Center for Information Technology, National Institute of Health. Gene symbol or blast search name, human unigene or gene bank accession number, chromosomal localization, predicted protein size and possible functions are shown. aa indicates amino acids. ^b^(–) indicates not found.

Table-5: Comparison of expression of some known ES marker by microarray and MPSS

| Gene | MPSS | Microarray | Microarray |
| --- | --- | --- | --- |
| Undifferentiated cell markers | PES/PEB^b^ | PES/PEB^a^ | BG02/Day 13/Day 21^a^ |
| DNMT3B | 1274/56 | 34/- | 22/2/5 |
| *POU5F1* | 658/20 | 23/3 | 28/3/4 |
| *GJA1* | 452/406 | 6/2 | 10/4/3 |
| *Galanin* | 221/28 | 5/4 | 33/4/3 |
| *NANOG* | 16/- | 8/- | 6/0/9 |
| *LEFTB* | 72/- | 24/- | 18/-/- |
| *TDGF1* | 38/- | 7/2 | 5/0/2 |
|  |  |  |  |

^a^ Indicates fold expression by microarray analysis compared to HuURNA.

^b^ Indicates transcripts per million (tpm) by MPSS analysis**.**

Table-6: Genes showed overexpression by microarray and EST

Enumeration in pooled samples of EB compared to pooled samples of ES

| Gene name | Accession number | EST-enumeration PES/PEB**^a^** | Microarray  PES/ PEB**^b^** |
| --- | --- | --- | --- |
| EB specific markers |  |  |  |
| *KRT8* | X74929 | 9/71 | 4/27 |
| *KRT18* | M26326 | 8/46 | 5/23 |
| *TUBB-5* | AK001295 | 5/8 | 6/11 |
| *ACTC* | J00073 | 1/4 | 4/6 |
| *TUBB4* | AK001295 | ND | 4/7 |
| Cell cycle regulation |  |  |  |
| *IMP-2* | NM_006548 | 9/14 | 5/7 |
| *NME2* | [M36981](http://www.ncbi.nlm.nih.gov/htbin-post/Entrez/query?db=n&form=6&uid=M36981&Dopt=g) | 3/6 | 3/8 |
| Metabolism DNA RNA related |  |  |  |
| *RPLP0* | AK001313 | 154/167 | 7/9 |
| *EIF4A1* | D13748 | 31/36 | 4/6 |
| *RPL24* | M94314 | ND^c^ | 4/6 |
| *RPL4* | [D23660](http://www.ncbi.nlm.nih.gov/htbin-post/Entrez/query?db=n&form=6&uid=D23660&Dopt=g) | ND | 3/10 |
| *SNRPF* | [X85372](http://www.ncbi.nlm.nih.gov/htbin-post/Entrez/query?db=n&form=6&uid=X85372&Dopt=g) | ND | 3/6 |
| *HMGIY* | [L17131](http://www.ncbi.nlm.nih.gov/htbin-post/Entrez/query?db=n&form=6&uid=L17131&Dopt=g) | ND | 9/15 |
| Metabolic activity |  |  |  |
| *SERPINH1* | D83174 | 28/32 | 4/10 |
| *CCT8* | D13627 | 8/10 | 4/5 |
| *SMS* | AD001528 | 5/7 | 4/5 |
| Novel genes |  |  |  |
| *C15orf15* | [NM_016304](http://www.ncbi.nlm.nih.gov/htbin-post/Entrez/query?db=n&form=6&uid=NM_016304&Dopt=g) | 2/6 | 4/7 |
| *TD-60* | [AB040903](http://www.ncbi.nlm.nih.gov/htbin-post/Entrez/query?db=n&form=6&uid=AB040903&Dopt=g) | ND | 3/7 |
| *C20orf168 / HNRPA1* | [AL050348](http://www.ncbi.nlm.nih.gov/htbin-post/Entrez/query?db=n&form=6&uid=AL050348&Dopt=g) | ND | 5/7 |
| *Numatrin* | AL353580 | ND | 4/7 |

^a^ Ratio of fold expression in pooled samples of ES and EB by EST enumeration

^b^ Indicates gene expression ratio of Pooled samples of ES and EB by microarray.

^c^ ND, expression not detected.

Table-7: Genes overexpressed by MPSS, microarray and EST enumeration in

Day 13 EB compared to BG02-ES

| Gene name | Accession number | EST-enumeration PES/PEB^a^ | Microarray BG02-ES / Day 13EB^b^ | MPSS  BG02-ES/ Day 13EB^c^ |
| --- | --- | --- | --- | --- |
| EB specific markers |  |  |  |  |
| *KRT8* | X74929 | 9/71 | 17/45 | 594/2472 |
| *KRT18* | M26326 | 8/46 | 23/24 | 323/2090 |
| *TUBB-5* | AK001295 | 5/8 | 5/8 | ND |
| *ACTC* | J00073 | 1/4 | 22/23 | 112/141 |
| *TUBB4* | AK001295 | NE^d^ | 4/5 | ND^e^ |
| Cell cycle regulation |  |  |  |  |
| *PITX2* | AF048722 | NE | 7/11 | ND |
| Metabolism DNA RNA related |  |  |  |  |
| *RPLP0* | AK001313 | 154/167 | 7/8 | ND |
| Metabolic activity |  |  |  |  |
| *SERPINH1* | D83174 | 28/32 | 6/8 | ND |

^a^ Indicates EST enumeration ratio of pooled samples of ES and EB.

^b^ Indicates gene expression ratio of Pooled samples of ES and EB by microarray

^c^ Indicates tpm level by MPSS analysis in BG02 ES and day 13 EB.

^d^ NE indicates no expression observed by EST enumeration.

^e^ ND indicates not done.

Table-8: Comparison of expression profile of 8 unknown genes by MPSS and microarray

| **Gene** | **Description** | **MPSS**  **ES /EB (tpm)^a^** | **Microarray PES/PEB ^b^** | **Microarray**  **BG02-ES/D-13 EB/ D21-EB ^b^** |
| --- | --- | --- | --- | --- |
| *FLJ10134* | Hypothetical protein FLJ10134 | 12/145 | -/3 | -/3/2 |
| *FLJ12541* | Hypothetical protein FLJ12541 | 0/26 | -/3 | -/3/- |
| *FLJ22329* | Hypothetical protein FLJ22329 | 5/86 | -/4 | 2/3/2 |
| *LOC348262* | Hypothetical proteins LOC348262 | 51/103 | -/3 | -/3/2 |
| *MDS025* | Hypothetical protein MDS025 | 9/15 | 2/5 | 2/3/2 |
| *MGC35097* | Hypothetical protein MGC35097 | 0/17 | -/4 | -/3/2 |
| *MGC51082* | Hypothetical protein MGC51082 | 18/40 | -/3 | -/3/2 |
| *MGC8721* | Hypothetical protein MGC8721/ XTP-3 / FLJ22274 | 83/101 | -/3 | -/3/- |

^a^ indicates the expression of these genes in pooled ES and EB by MPSS analysis at tpm level. ^b^ indicates the fold expression of these genes in both ES and EBs by microarray.

(-) indicates ≤ 1 fold or no expression.

Table-9: Categorization of 46 genes expressed uniquely in Day 13 EB and pooled samples of EB by microarray but not by MPSS^a^

| Category^b^ | Gene |
| --- | --- |
| ES cell differentiation specific genes (1) | TWSG1 |
| Cellular process (Cell signaling, Cell growth, Cellular process, Cell cycle) (11) | DDX43, *ERBB2*, *GPR43*, *HIVEP3*, *IL26, KCNA5*, *MYBL2*, *NID2*, *RAB27A*, *SLC9A3R1*, *TRIM34* |
| Cytoskeleton or cell motility (2) | *SPTA1*, *TUBA4* |
| Metabolic activity  DNA and RNA related (2) | *KIAA1318*, *RPL18* |
| Metabolism (21) | *ARHGAP9*, C5ORF15, *C11ORF9, C14ORF147*, *CHST2*, *DSCR4*, *DXYS155E*, GBA, *GOLGA6, LEPREL2*, LOC347544, LOC51215, MCM8, *PRKWNK3*, PRO1776, PRO2792, PRO2852, *S100A11*, *UBE2E3*, *VNN3, VTN* |
| Unknown genes (9) | *ZNF306*, ZNF350, *FLJ12650*, FLJ12983, FLJ21031, *FLJ23233*  *H1FX*, *POF1B*, *ZP2* |

^a^Genes expressed at 99% confidence interval (≥ 3 fold) are shown. Expression of the genes in italics is confirmed by Agilent human 22k oligo-array (G4110B). ^b^Numbers in parenthesis represent the number of genes under each category.

Table-10: Gene expression in Day 13 EB and Day 21 EB^a^

| Gene | BG02-ES | Day 13 BG02-EB | Day 21 BG02-EB |
| --- | --- | --- | --- |
| *KRT8* | 17 | 45 | 1 |
| *KRT18* | 23 | 24 | 5 |
| *TUBB5* | 5 | 8 | 2 |
| *ACTC* | 22 | 23 | 1 |
| *RPLP0* | 7 | 8 | 6 |
| *SERPINH1* | 6 | 8 | 1 |
| *TUBB4* | 4 | 5 | 2 |
| *RPS24* | 15 | 17 | 17 |

^a^ Indicates fold expression compared to HuURNA.
